# Multifunctional Manganese Oxide Nanocomposite Hydrogel With Synergistic Reactive Oxygen Species‐Scavenging, Oxygen‐Generating, Immunomodulatory, and Photothermal Antimicrobial Activities for Enhanced Diabetic Wound Healing

**DOI:** 10.1002/advs.76774

**Published:** 2026-07-24

**Authors:** Zhi Xu, Xiaodong Luo, Jingxian Wu, Chaoyang Huang, Zhuoying Yang, Yixiang HePeng, Bosong Zhou, Xiang Li, Ruiyuan Liu, Xu Wu

**Affiliations:** ^1^ Department of Thoracic Surgery Nanfang Hospital Southern Medical University Guangzhou China; ^2^ Huiqiao Medical Center Nanfang Hospital Southern Medical University Guangzhou China; ^3^ Department of General Practice Nanfang Hospital Southern Medical University Guangzhou China; ^4^ Cardiovascular Surgery the Second Affiliated Hospital of Kunming Medical University Kunming China; ^5^ Department of Burns Nanfang Hospital Southern Medical University Guangzhou China; ^6^ Department of Emergency Medicine Nanfang Hospital Southern Medical University Guangzhou China; ^7^ Biomaterials Research Center, School of Biomedical Engineering Southern Medical University Guangzhou China

**Keywords:** alleviating hypoxia, antioxidative stress, photothermal antibacterial, self‐healing hydrogel, wound healing

## Abstract

Diabetic wounds are characterized by a complex pathological microenvironment, marked by persistent hypoxia, excessive reactive oxygen species (ROS), chronic inflammation, and bacterial infection. To address this challenge, we developed a multifunctional composite hydrogel (CFP/PC@MnO_2_) for diabetic wound healing. It is formed by covalent crosslinking of carboxymethyl chitosan (CMCS), 4‐formylphenylboronic acid (4FPBA), and polyvinyl alcohol (PVA) via Schiff base and boronate ester bonds, followed by incorporation of phycocyanin‐modified MnO_2_ nanoparticles (PC@MnO_2_ NPs). The CFP/PC@MnO_2_ hydrogel exhibits favorable mechanical properties, local injectability, self‐healing capability, tissue adhesion, and the ability to form a protective wound barrier. Meanwhile, the CFP/PC@MnO_2_ hydrogel degrades rapidly under ROS, releasing PC@MnO_2_ to scavenge ROS and generate O_2_ in situ to alleviate hypoxia; the PC@MnO_2_ NPs further exhibit strong NIR‐triggered photothermal antibacterial activity. In vitro, this hydrogel exhibits excellent biocompatibility, effectively scavenges ROS, alleviates oxidative stress, and suppresses inflammation. In a full‐thickness wound model in diabetic rats, the CFP/PC@MnO_2_ hydrogel combined with photothermal therapy effectively eradicates bacteria, promotes M2 macrophage polarization to modulate the immune microenvironment, and enhances angiogenesis and collagen deposition, thereby accelerating wound healing. Overall, this multifunctional hydrogel integrates ROS scavenging, self‐sustained oxygenation, immunomodulation, and photothermal antibacterial action, offering a synergistic, innovative strategy for diabetic wound treatment.

## Graphical abstract

CFP/PC@MnO_2_ hydrogel integrates ROS scavenging, oxygen delivery, oxidative stress reduction, immunomodulation, and photothermal antibacterial properties. The incorporation of PC@MnO_2_ NPs endows the hydrogel with multiple functional advantages tailored to the pathological microenvironment of diabetic wounds, including efficient ROS clearance, mitigation of local hypoxia, modulation of immune‐inflammatory responses, promotion of M2 macrophage polarization, prophylactic antibacterial protection, and promotion of angiogenesis.

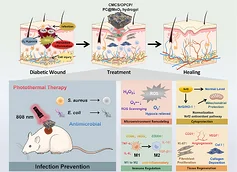

## 1 Introduction

Diabetes mellitus (DM) is a chronic metabolic disorder marked by sustained hyperglycemia, impacting hundreds of millions of people worldwide [1, 2]. Its prevalence is escalating at a concerning pace, positioning it as a significant public health challenge worldwide. The 11th edition of the International Diabetes Federation (IDF) Diabetes Atlas reports that the global prevalence of diabetes among adults was 11.1% (589 million) in 2024, with projections indicating an increase to 12.96% (853 million) by 2050 [3]. Long‐term diabetic complications influence multiple organ systems, including diabetic foot ulcers, nephropathy, retinopathy, atherosclerosis, and neuropathy [4]. Among these complications, diabetic foot ulcers represent a substantial clinical challenge due to their complex pathophysiology, chronic nature, and high rates of recurrence [5]. The pathological microenvironment of diabetic wounds is characterized by excessive oxidative stress, persistent inflammation, overproduction of reactive oxygen species (ROS), pronounced tissue hypoxia, chronic infection, and dysfunction of key regenerative cells [6, 7, 8, 9]. These interconnected factors establish a self‐perpetuating cycle that hinders wound healing and results in chronic, non‐healing wounds. Therefore, the development of multifunctional bioactive dressings capable of precisely modulating the diabetic wound microenvironment is essential to facilitate the healing of chronic wounds.

Tissue hypoxia and excessive accumulation of ROS represent two pivotal pathological factors that synergistically contribute to impaired wound healing in diabetic conditions. Hypoxia not only directly impairs epithelial regeneration, angiogenesis, and extracellular matrix synthesis, but also intensifies oxidative stress by inhibiting antioxidant defense mechanisms and inducing mitochondrial dysfunction [10, 11, 12]. The overabundance of ROS, such as hydrogen peroxide (H_2_O_2_) and superoxide anions (O_2_^•−^), further damages cellular integrity and function through processes including lipid peroxidation, protein inactivation, and DNA damage [13, 14, 15]. This systemic dysregulation of the balance between hypoxia and oxidative stress not only amplifies inflammatory responses but also significantly hinders tissue repair and regeneration. Consequently, simultaneously alleviating hypoxia and scavenging excess ROS are essential strategies to facilitate wound healing.

Manganese dioxide (MnO_2_) nanozymes possess bifunctional enzymatic activities, effectively emulating both superoxide dismutase (SOD) and catalase (CAT) enzymes [16, 17, 18, 19]. Specifically, MnO_2_ nanozymes catalyze the dismutation of O_2_^•−^ into O_2_ and H_2_O_2_, and further decompose H_2_O_2_ into H_2_O and O_2_. This sequential catalytic process facilitates concurrent ROS scavenging and oxygen generation, thereby offering extensive potential for various biomedical applications. Beyond their enzyme‐mimetic catalytic functions, MnO_2_ NPs exhibit strong absorption in the near‐infrared (NIR) spectrum, endowing them with exceptional photothermal conversion capability [20, 21]. This property enables the effective conversion of light energy into heat for photothermal antibacterial therapy. MnO_2_ nanozymes serve as pivotal active components in diabetic wound dressings, owing to their capacity to alleviate hypoxia, scavenge ROS, and exert photothermal antibacterial effects. For example, Wang et al. encapsulated polydopamine (PDA) and MnO_2_ into a Dex‐SA‐AEMA hydrogel to create a multifunctional platform that eradicates biofilms, generates oxygen, and exerts photothermal antibacterial effects for chronic diabetic wound healing [22]. Guo et al. developed an in situ‐forming, antibacterial, and antioxidant SF@(EPL‐BM) hydrogel by combining methacrylated silk fibroin (SFMA), ε‐polylysine (EPL), and MnO_2_ nanoparticles (BMNP), showing promising therapeutic potential in diabetic mice [23]. Jin et al. developed a sprayable hydrogel (MnO_2_@COF–GOx/Gel) by integrating COF‐encapsulated MnO_2_ with GOx and methacrylated alginate gel, promoting diabetic chronic wound healing via ROS scavenging, glucose regulation, and antibacterial activity [24]. Nevertheless, the intrinsic hydrophobicity of MnO_2_ and the limited availability of reactive functional groups on its surface restrict its colloidal stability in aqueous environments and impede further chemical modification [25]. Phycocyanin (PC), a water‐soluble protein derived from algae, demonstrates a high affinity for cations and functions as an effective stabilizing agent during nanoparticle synthesis and surface coating processes [26, 27, 28, 29, 30]. Moreover, PC contains a linear tetrapyrrole chromophore structurally analogous to bilirubin, which endows it with potent antioxidant and anti‐inflammatory properties [31, 32, 33]. These characteristics contribute to the mitigation of oxidative stress, rendering PC highly promising for applications in diabetic wound management and playing a pivotal role in counteracting hyperglycemia‐induced tissue damage [34, 35, 36]. Therefore, the development of a novel nanoplatform through the modification of MnO_2_ with phycocyanin offers a multifaceted therapeutic strategy that combines photothermal antibacterial activity with the alleviation of tissue hypoxia and oxidative stress, ultimately facilitating tissue regeneration in diabetic wounds.

The development of a controllable delivery system is essential to harness the therapeutic potential of nanozymes [37, 38]. In the absence of such a system, nanozymes undergo burst release, which prevents them from maintaining effective therapeutic concentrations within the critical timeframe necessary for wound healing. Hydrogels are considered one of the most promising delivery platforms owing to their three‐dimensional network structure, which provides a moist microenvironment and facilitates efficient loading as well as sustained release of bioactive agents [39, 40, 41]. Notably, the incorporation of reversible covalent bonds (such as imine and boronate ester bonds) not only endows hydrogels with excellent injectability, self‐healing capability, and tissue adhesiveness, but also confers ROS‐ and pH‐responsive drug release properties, thereby better meeting the therapeutic requirements for irregular and diabetic wounds [42, 43, 44].

Based on the above analysis, we have developed a multifunctional composite hydrogel specifically aimed at enhancing diabetic wound healing. This hydrogel integrates ROS scavenging, oxygen delivery, oxidative stress reduction, immunomodulation, and photothermal antibacterial activity, designated as the CFP/PC@MnO_2_ hydrogel. This hydrogel is composed of CMCS, 4FPBA, PVA, and phycocyanin‐modified MnO_2_ (PC@MnO_2_ NPs), forming a robust crosslinked network via dynamic Schiff base and boronate ester bonds, thereby exhibiting excellent mechanical properties (Scheme 1A). The incorporation of PC@MnO_2_ NPs endows the hydrogel with multiple functional advantages tailored to the pathological microenvironment of diabetic wounds, including efficient ROS clearance, mitigation of local hypoxia, modulation of immune‐inflammatory responses, promotion of M2 macrophage polarization, prophylactic antibacterial protection, and promotion of angiogenesis (Scheme 1B). Collectively, this study establishes a rationally designed multifunctional hydrogel platform that offers a novel and synergistic therapeutic approach for the treatment of chronic diabetic wounds.

**SCHEME 1:**
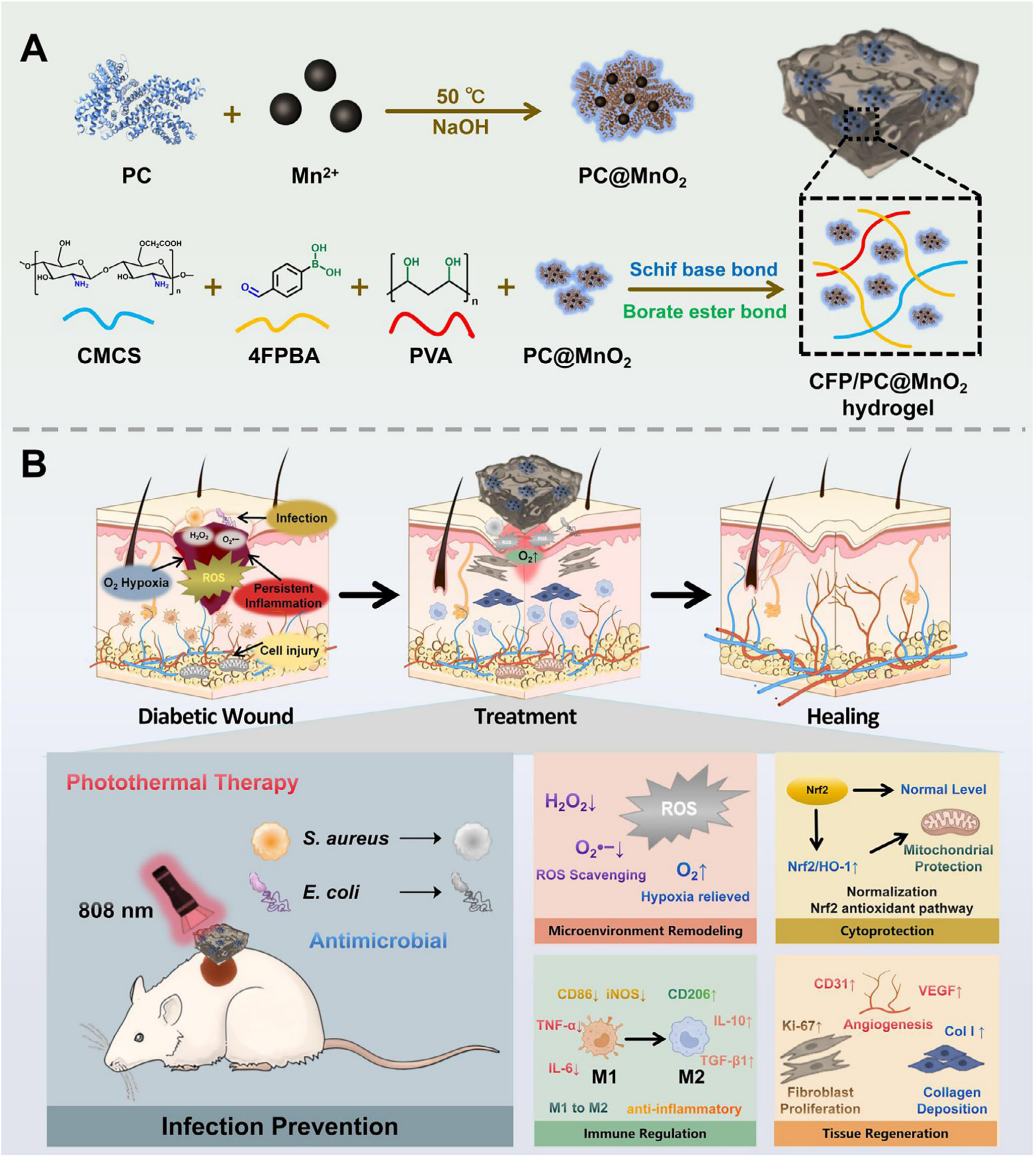
The preparation process and multimodal strategies to improve diabetic wound healing using the CFP/PC@MnO_2_ hydrogel. (A) Preparation of the dynamic cross‐linked CFP/PC@MnO_2_ hydrogel network. (B) Application in diabetic wound healing.

## 2 Experimental Section

### 2.1 Synthesis and Characterization of PC@MnO_2_ NPs

1 mL of 0.2 M MnCl_2_ solution was added to 18 mL of PC solution (25 mg mL^−1^) at 50°C, followed by vigorous stirring for 15 min. Subsequently, 1 mL of 1 M NaOH solution was rapidly introduced into the above mixture, and the reaction was allowed to proceed under continuous stirring for 2 h. The resulting product was then purified to afford PC@MnO_2_ NPs. The obtained PC@MnO_2_ NPs were characterized by DLS, zeta potential, TEM, XPS, and FT‐IR.

### 2.2 Preparation and Characterization of CFP Hydrogel and CFP/PC@MnO_2_ Hydrogel

CMCS, 4FPBA, PVA, and PC@MnO_2_ NPs were mixed at room temperature in predetermined mass ratios. A homogeneous precursor mixture was prepared by equal‐volume mixing at room temperature of a 5% (w/v) aqueous CMCS solution with a mixed solution containing 2% 4FPBA and varying amounts of PVA (0%, 3%, 6%, or 10%). The mixture was then poured into molds and allowed to stand to facilitate hydrogel formation, yielding CFP hydrogels named C5F2P3, C5F2P6, and C5F2P10. Subsequently, PC@MnO_2_ NPs were incorporated into each hydrogel system to achieve a final concentration of 400 µg mL^−1^, affording the CFP/PC@MnO_2_ hydrogel. The cross‐sectional morphology and elemental distribution of both the pristine CFP hydrogel and the CFP/PC@MnO_2_ hydrogel were systematically characterized using SEM coupled with EDS.

### 2.3 Evaluation of Extracellular Oxygen Generation and Antioxidant Capacity of PC@MnO_2_ NPs

The oxygen‐producing capability of PC@MnO_2_ NPs was measured using a commercial dissolved oxygen probe (AR8210). Briefly, 100 µg mL^−1^ PC@MnO_2_ NPs were mixed with 20 mM H_2_O_2_, and the dissolved oxygen concentration was monitored continuously for 10 min. H_2_O_2_, O_2_•^−^, and ABTS^+^• scavenging assays were used to evaluate ROS clearance capacities. For specific details, please refer to the Supporting Information.

### 2.4 Evaluation of Photothermal Properties of PC@MnO_2_ NPs

The photothermal performance of PC@MnO_2_ NPs was systematically evaluated under 808 nm laser irradiation. For concentration dependence, solutions (100–400 µg mL^−1^) were irradiated at 2.0 W/cm^2^ for 10 min. For power dependence, a fixed concentration (400 µg mL^−1^) was irradiated at 0.5–2.0 W/cm^2^ for 10 min. Photothermal stability was assessed over four on/off cycles (10 min irradiation/10 min cooling) at 2.0 W/cm^2^. Real‐time temperature changes were monitored using an infrared thermal imager (Fluke TiS20). Photothermal conversion efficiency was calculated from the temperature rise of the NP solution under 808 nm irradiation (2.0 W/cm^2^) until steady state (600 s). η = [*h*A(T_max_− T_surr_)—Q_Dis_]/[I(1‐ 10^−A^_λ_)] [45].

### 2.5 Evaluation of Intracellular ROS Elimination of CFP/PC@MnO_2_ Hydrogel

L929 and HUVEC cells were seeded in 48‐well plates at 5 × 10^4^ cells well^−1^ and cultured for 24 h. Then, 100 µL of hydrogel was added for co‐culture for another 24 h, followed by 2 h treatment with 400 µM H_2_O_2_ to induce oxidative stress. Cells were then incubated with serum‐free medium containing 10 µM DCFH‐DA for 30 min in the dark. Finally, fluorescence microscopy imaging was performed to evaluate the ROS scavenging capability.

### 2.6 Evaluation of Intracellular O2‐Generation Capacities of CFP/PC@MnO_2_ Hydrogel

Oxygen generation capacity of hydrogels inside cells was assessed using the [Ru(dpp)_3_]Cl_2_ probe. L929 cells were seeded into 48‐well plates at a density of 5 × 10^4^ cells per well. Subsequently, 100 µL of hydrogel was added to each well, and the plates were incubated for 24 h under oxidative conditions containing 100 µM hydrogen peroxide (H_2_O_2_). Thereafter, [Ru(dpp)_3_]Cl_2_ (10 µg mL^−1^) was added, and incubation was continued for an additional 12 h. Finally, cellular fluorescence was visualized using a fluorescence microscope.

### 2.7 Modulates Macrophage Polarization via CFP/PC@MnO_2_ Hydrogel

RAW 264.7 macrophages were seeded at optimal density in confocal dishes, co‐cultured with hydrogels, and then stimulated with LPS (for M1) or IL‐4 (for M2). After incubation, cells were washed with PBS and subjected to fluorescence staining for M1/M2 markers (CD86, iNOS, and CD206).

### 2.8 Anti‐Inflammatory Capacity of CFP/PC@MnO_2_ Hydrogel

RAW 264.7 macrophages were seeded at an appropriate density onto confocal dishes and co‐cultured with hydrogels. Subsequently, cells were stimulated with either LPS to induce TNF‐α or IL‐10 expression, respectively. After incubation, the cells were washed with PBS and then incubated sequentially with primary antibodies specific for TNF‐α and IL‐10, followed by corresponding fluorescently labeled secondary antibodies. Finally, protein expression was visualized and recorded using confocal microscopy.

### 2.9 Antibacterial Activity Evaluation of CFP/PC@MnO_2_ Hydrogel

This study employed *Staphylococcus aureus* (*S. aureus*) and *Escherichia coli* (*E. coli*) as model microorganisms to evaluate the antibacterial efficacy of the hydrogels. In a 48‐well plate, 1 mL of bacterial suspension (1 × 10^5^ CFU mL^−1^) was added to each well and co‐cultured for 12 h at 37°C with either CFP hydrogel, CFP/PC@MnO_2_ hydrogel, or PC@MnO_2_ NPs (400 µg mL^−1^). For the photothermal therapy group, samples were irradiated with an 808 nm laser at a power density of 2.0 W/cm^2^ for 10 min. Subsequently, 100 µL of each bacterial suspension was plated onto LB agar plates and incubated at 37°C for 24 h. Colony‐forming units (CFUs) were enumerated, and the antibacterial rate was calculated using the following formula: Antibacterial rate (%) ─ (A_1_ − A_2_)/A_1_ × 100%, where A_1_ represents the mean CFU count of the control group and A_2_ denotes the mean CFU count of the respective treatment group. To assess bacterial viability, aliquots of treated bacterial suspensions were stained with SYTO‐9 and PI for 15 min, mounted on glass slides, and visualized under a fluorescence microscope to distinguish live from dead cells. Bacterial morphology was further examined by SEM. Additionally, crystal violet staining was performed to quantitatively evaluate the biofilm removal efficiency of the hydrogels.

### 2.10 In Vivo Wound Healing Assay

All animal experiments were conducted in accordance with protocols approved by the local ethics committee. Thirty 7‐week‐old male SD rats (purchased from the Animal Center of Southern Medical University) were randomly assigned to five groups: control, PC@MnO_2_ NPs, CFP hydrogel, CFP/PC@MnO_2_ hydrogel, and CFP/PC@MnO_2_ hydrogel + NIR (= 6). Type 1 diabetes was induced by intraperitoneal injection of streptozotocin (STZ; 70 mg kg^−1^) after 1 week of acclimatization and overnight fasting [46]. Animals with sustained fasting blood glucose > 16.7 mM and polydipsia, polyphagia, and polyuria within 1 week were considered successfully induced. After induction, rats were anesthetized with 2% sodium pentobarbital, shaved on the back, and subjected to six 10‐mm full‐thickness circular wounds. Wounds were treated with PBS, PC@MnO_2_ NPs, CFP hydrogel, or CFP/PC@MnO_2_ hydrogel, respectively; the NIR group received additional 808 nm laser irradiation (2.0 W/cm^2^). All wounds were covered with gauze and secured with 3 M film. Rats were housed individually. Wound images were captured on days 0, 3, 7, and 15 post‐treatment to calculate wound closure rates. Each rat served as an independent experimental unit. For each rat, the mean wound measurement across all wounds at each time point was calculated as the animal's representative value, followed by intergroup comparisons. On day 4, wound tissues were harvested to assess bacterial load.

### 2.11 Tissue Staining

On days 7, 10, and 15 of treatment, one wound per rat was harvested, fixed in 4% paraformaldehyde, paraffin‐embedded, and serially sectioned for H&E staining, Masson's trichrome staining, immunohistochemical detection of TNF‐α, IL‐6, TGF‐β1, VEGF, CD31, and Ki‐67, and immunofluorescent detection of DHE, HIF‐1α, CD86, and CD206 to assess wound healing, collagen deposition, inflammatory regulation, angiogenesis, and cell proliferation. At study termination, heart, liver, spleen, lung, and kidney tissues were collected for histopathological evaluation to assess biosafety.

### 2.12 Statistical Analysis

This study used OriginPro 2025 (OriginLab, USA) for statistical analysis. Continuous data are presented as mean ± standard deviation. Differences among multiple groups were assessed by one‐way ANOVA with Tukey's multiple comparison test. A *p* value < 0.05 was considered statistically significant.

## 3 Results and Discussion

### 3.1 Synthesis and Characterization of PC@MnO_2_ NPs

As shown in Figure 1A, the preparation strategy of PC@MnO_2_ NPs is achieved through a biomineralization process. Under mild temperature conditions, the PC solution and MnCl_2_ solution are thoroughly mixed to facilitate the coordination of Mn^2^^+^ ions with active functional groups of PC, such as carboxyl and thiol groups. The pH was subsequently adjusted to facilitate the formation of phycocyanin‐coated manganese oxide nanoclusters. Subsequently, the UV‐vis absorption spectrum of the PC@MnO_2_ NPs was recorded (Figure 1B). Compared with PC, a novel absorption band emerged within the 300 ∼ 400 nm, which is attributed to the surface plasmon resonance characteristic of manganese dioxide. Additionally, pronounced absorption was detected between 720 and 900 nm, suggesting their potential for NIR photothermal conversion applications. Characterization via dynamic light scattering (DLS) and transmission electron microscopy (TEM) demonstrated that the synthesized PC@MnO_2_ NPs possess a uniform particle size distribution with an average particle size of approximately 146.85 nm (Figure 1C). As shown in Figure 1D, PC and PC@MnO_2_ NPs exhibited negative surface potentials of −26.20 and −13.36 mV, respectively. The reduction in the surface potential of PC@MnO_2_ NPs confirmed the successful loading of manganese oxide nanoparticles onto the PC. X‐ray photoelectron spectroscopy (XPS) was utilized to investigate the chemical valence state of manganese in PC@MnO_2_. Two distinct characteristic peaks were detected at 642.5 eV and 654.3 eV, which are assigned to the Mn 2p1/2 and Mn 2p3/2 spin‐orbit doublets of MnO_2_, respectively, confirming that manganese is present in the +4 oxidation state (Figure 1E). Fourier transform infrared spectroscopy (FT‐IR) further revealed strong peaks at 1150 and 2925 cm^−1^ in PC@MnO_2_ NPs, which are attributed to the C‐O‐C stretching vibrations of glycosidic bonds between sugar units and the C‐H stretching vibrations, respectively, thereby confirming the presence of phycocyanin in the PC@MnO_2_ NPs (Figure S1). Next, the nanostability of PC@MnO_2_ NPs stored in PBS and DMEM solutions for 7 days was evaluated. As shown in Figures S2 and S3, no significant changes in hydrodynamic diameter were observed, confirming their favorable stability. The above results fully demonstrate the successful preparation of PC@MnO_2_ NPs and their excellent stability.

**FIGURE 1:**
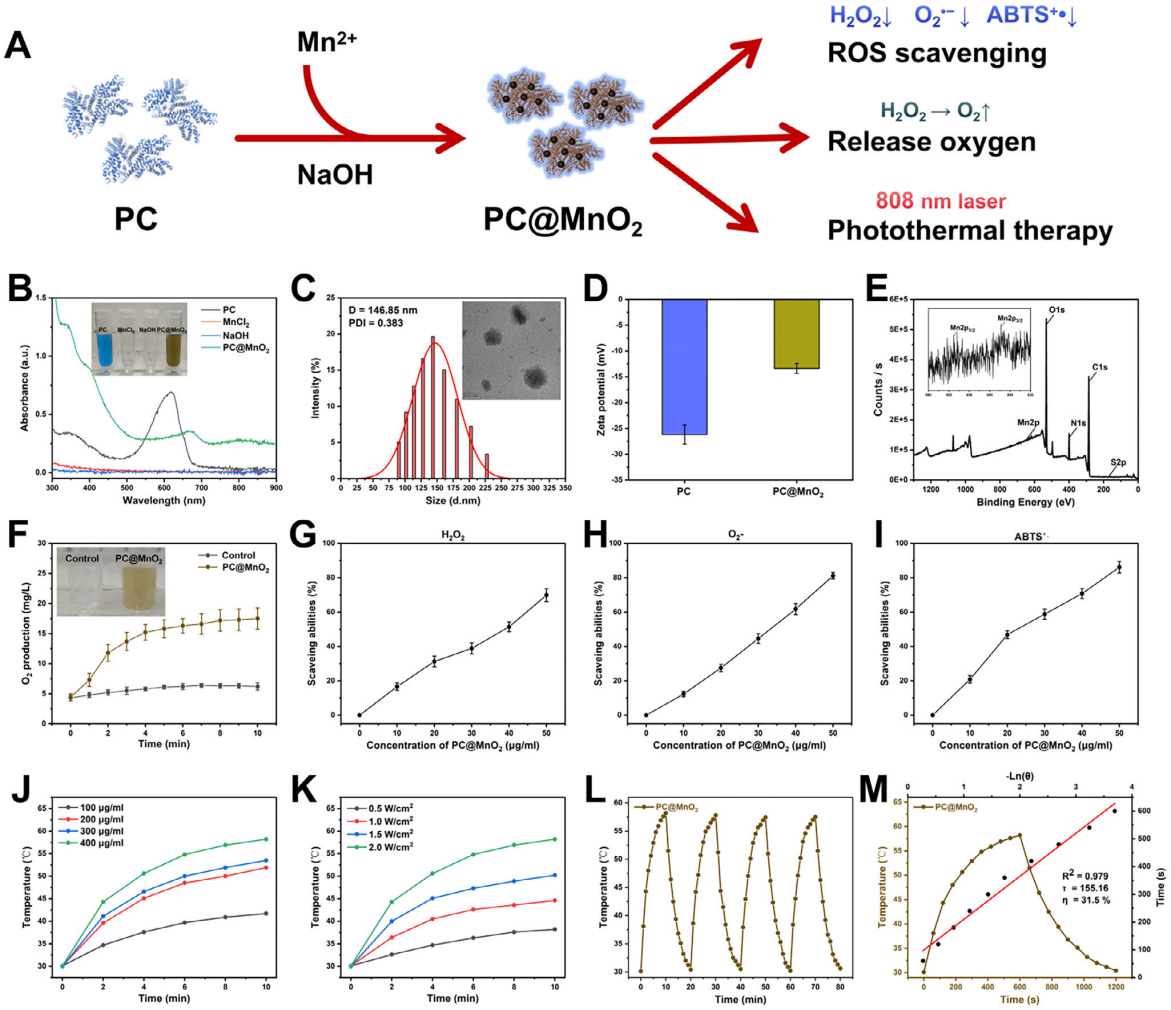
The characterization of PC@MnO_2_ NPs. (A) Synthetic strategy for PC@MnO_2_ NPs. (B) UV–vis‐NIR absorption spectra of PC@MnO_2_ NPs. (C) TEM and DLS size distribution of PC@MnO_2_ NPs. (D) Zeta potential of PC and PC@MnO_2_ NPs. Data are expressed as mean ± standard deviation. (*n* = 3). (E) The XPS spectrum of PC@MnO_2_ NPs. Oxygen generation and antioxidant capacity of PC@MnO_2_ NPs. (F) O_2_ production ability of PC@MnO_2_ NPs. Data are expressed as mean ± standard deviation. (*n* = 3). (G‐I) Radical scavenging efficiency of PC@MnO_2_ NPs. Data are expressed as mean ± standard deviation. (*n* = 3). Data are expressed as mean ± standard deviation. (*n* = 3). The Photothermal Properties of PC@MnO_2_ NPs. (J) Temperature profiles of PC@MnO_2_ NPs at varying concentrations under 808 nm laser irradiation (2.0 W/cm^2^). (K) Temperature profiles of 400 µg mL^−1^ PC@MnO_2_ NPs under 808 nm laser irradiation at different power densities. (L) Photothermal stability of PC@MnO_2_ NPs during four on–off laser irradiation cycles (400 µg mL^−1^; 2.0 W/cm^2^). (M) Photothermal conversion efficiency profile of PC@MnO_2_ NPs.

### 3.2 Oxygenation and Antioxidation of PC@MnO_2_ NPs

The healing of chronic wounds is severely constrained by a hypoxic microenvironment. Concurrently, the excessive accumulation of ROS in this environment can lead to uncontrolled inflammatory responses and tissue damage [47]. Hence, a therapeutic approach that combines both oxygen‐generating and antioxidant functionalities is expected to substantially improve the healing process of chronic wounds through a synergistic mechanism. To emulate the oxidative microenvironment characteristic of diabetic wounds, a working solution comprising 10 mM hydrogen peroxide (H_2_O_2_) and 50 µg mL^−1^ PC@MnO_2_ NPs was employed, with dissolved oxygen levels monitored over a 10 min interval. As depicted in Figure 1F, the concentration of dissolved oxygen increased progressively over time, ultimately attaining 17.5 mg L^−1^. Notably, pronounced bubble formation was observed, attributable to the catalase‐mimetic enzymatic activity of manganese dioxide [17]. Subsequently, the ROS scavenging efficacy of PC@MnO_2_ NPs was further assessed against H_2_O_2_, superoxide anion radicals (O_2_•−), and ABTS^+^• radicals. When the concentration reached 50 µg mL^−1^, the scavenging efficiencies reached 69.87%, 81.15%, and 86.09%, respectively (Figure 1G–I and Figure S4). Collectively, these findings demonstrate that PC@MnO_2_ NPs possess robust oxygen‐generating and antioxidant properties, effectively catalyzing the conversion of hydrogen peroxide into oxygen to mitigate hypoxia, while concurrently scavenging ROS. These attributes underscore their considerable potential for enhancing wound healing in clinical contexts.

### 3.3 The Photothermal Properties of PC@MnO_2_ NPs

Owing to their superior near‐infrared absorption characteristics, the in vitro photothermal properties of PC@MnO_2_ NPs were thoroughly examined. Initially, the photothermal response of PC@MnO_2_ NPs was systematically assessed across a range of concentrations. As shown in Figure 1J, exposure of PC@MnO_2_ NPs at varying concentrations to a laser power density of 2.0 W/cm^2^ induced differential temperature elevations over a 10 min period. Notably, at a concentration of 400 µg mL^−1^, the temperature increased rapidly from 30.1°C to 58.2°C. Subsequently, the influence of varying laser power densities on the thermal response of PC@MnO_2_ NPs was investigated. Maintaining the nanoparticle concentration at 400 µg mL^−1^, continuous laser irradiation for 10 min at power densities of 0.5, 1.0, 1.5, and 2.0 W/cm^2^ resulted in final temperatures of 38.2°C, 44.6°C, 50.2°C, 58.2°C, respectively (Figure 1K). The photostability of PC@MnO_2_ NPs was further assessed through four consecutive on‐off laser irradiation cycles (400 µg mL^−1^; 2.0 W/cm^2^), during which the peak temperature attained in each cycle remained stable without significant fluctuation (Figure 1L). The photothermal conversion efficiency of PC@MnO_2_ NPs was calculated to be 31.5%. The aforementioned results confirmed that PC@MnO_2_ NPs exhibit excellent photothermal conversion efficiency and photothermal stability.

### 3.4 Synthesis and Characterization of CFP/PC@MnO_2_ Hydrogel

CFP hydrogel was formed by cross‐linking carboxymethyl chitosan (CMCS) and PVA with the crosslinker 4FPBA, while CFP/PC@MnO_2_ hydrogels were derived from the same system with the incorporation of PC@MnO_2_ NPs (Figure 2A). The primary cross‐linking mechanisms of the CFP hydrogel arise from Schiff base formation between the free amino groups of CMCS and the aldehyde groups of 4FPBA, as well as boronate ester bond formation between 4FPBA and PVA. FT‐IR spectra showed a broad peak at 3430 cm^−1^ for CMCS, assigned to overlapping O‐H and N‐H stretching vibrations; peaks at 1610 and 1427 cm^−1^ corresponded to asymmetric and symmetric stretching vibration absorption peaks of the ‐COOH group. 4FPBA showed a C═O stretching peak at 1670 cm^−1^ and a B─O─C asymmetric stretching peak at 1340 cm^−1^. PVA displayed an O─H stretching peak at 3400 cm^−1^ (Figure S5). In CFP and CFP/PC@MnO_2_ hydrogels, the C═O peak at 1670 cm^−1^ weakens, while a new C═N stretching peak appears at 1575 cm^−1^, confirming imine bond formation; additionally, a B‐O‐C stretching peak emerges at 1421 cm^−1^, indicating boronate ester cross‐linking between PVA and 4FPBA (Figure 2B).

**FIGURE 2:**
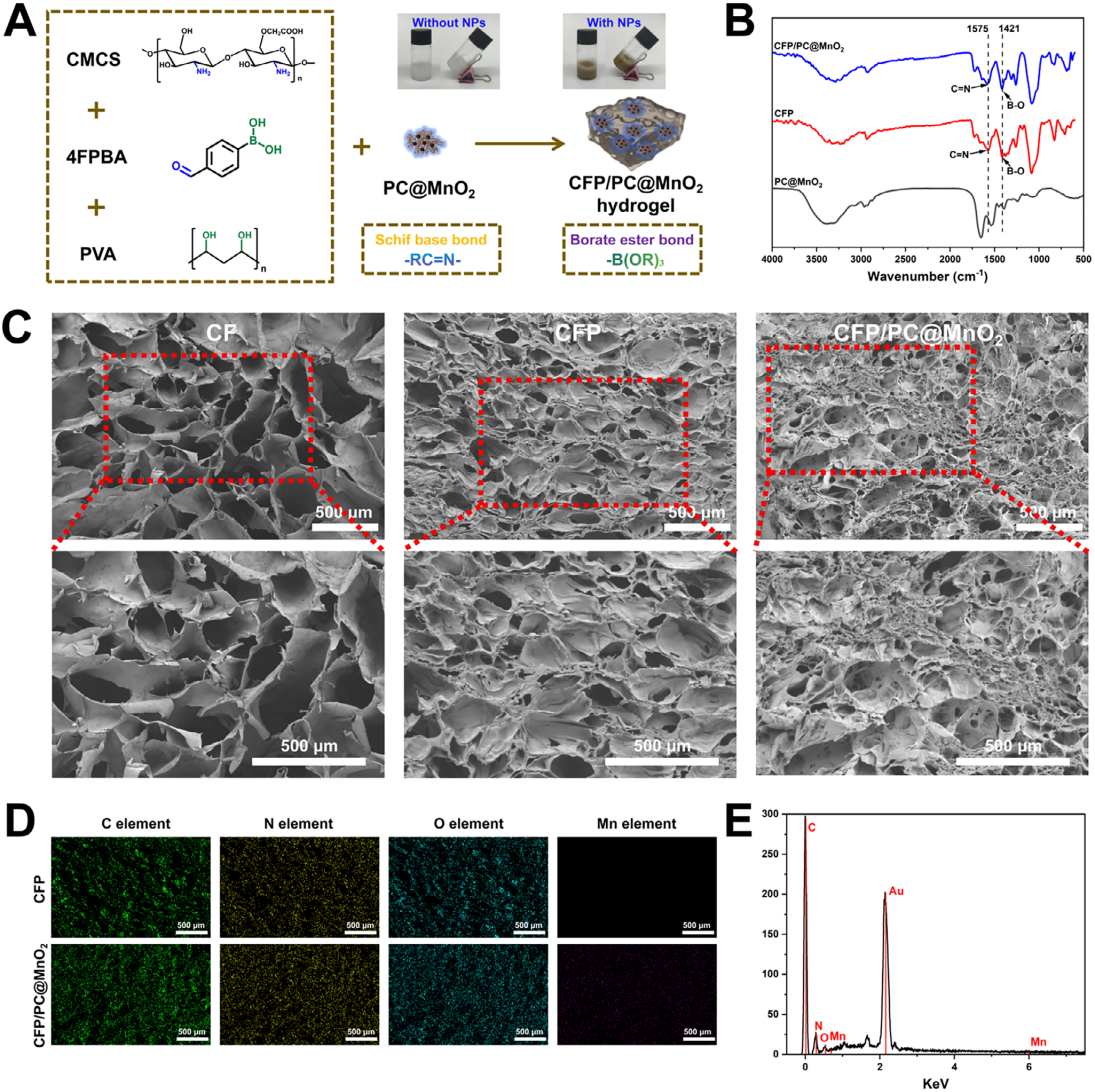
The characterization of CFP/PC@MnO_2_ hydrogel. (A) Strategies for synthesizing CFP/PC@MnO_2_ hydrogel. (B) The FT‐IR spectra of PC@MnO_2_, CFP and CFP/PC@MnO_2_ hydrogel. (C) The SEM images of CFP hydrogel and CFP/PC@MnO_2_ hydrogel. (D) Elemental mapping of C, N, O, and Mn in CFP/PC@MnO_2_ hydrogel. (E) EDS spectrum of CFP/PC@MnO_2_ hydrogel.

The gelation time of CFP hydrogels and CFP/PC@MnO_2_ hydrogels was investigated using the inverted vial method. Gelation time decreased significantly with increasing PVA content, attributed to additional hydroxyl groups from PVA enhancing boronate ester formation and thus accelerating gelation. Notably, incorporating PC@MnO_2_ nanoparticles into the CFP hydrogel did not significantly prolong gelation time (Figure S6). Further analysis was performed using SEM, revealing that both the CFP hydrogels and CFP/PC@MnO_2_ hydrogels exhibit well‐defined and uniformly distributed porous microstructures (Figure 2C). Moreover, increasing the PVA content in CFP hydrogels gradually densified their porous structure (Figure S7). Elemental analysis confirmed the homogeneous distribution of carbon, nitrogen, oxygen, and manganese, thereby verifying the successful incorporation of PC@MnO_2_ NPs into the CFP hydrogel matrix. The corresponding mass percentages of carbon, nitrogen, oxygen, and manganese were determined to be 41.75%, 21.43%, 35.47%, and 1.35%, respectively (Figure 2D,E).

### 3.5 Physicochemical Properties of CFP/PC@MnO_2_ Hydrogel

An appropriate elastic modulus in hydrogels ensures conformal wound support, structural integrity during exudate absorption, reduced discomfort, and enhanced healing [48]. We evaluated the mechanical properties of CFP hydrogels with varying PVA content. The compression modulus increased significantly with PVA content: Young's moduli of C5F2P0, C5F2P3, C5F2P6, and C5F2P10 were 23.63, 44.33, 68.51, and 98.28 kPa, respectively. Among them, C5F2P10 exhibited the optimal elastic modulus and was selected for subsequent tests (Figures S8 and S9). Effective management of wound exudate is critical for healing. Owing to their swelling behavior and high water‐absorption capacity, hydrogels efficiently remove exudate and create an optimal microenvironment for tissue repair [49]. As shown in Figure 3A, CF, CFP, and CFP/PC@MnO_2_ hydrogels all exhibited excellent swelling performance, reaching equilibrium within 3 h. The equilibrium swelling ratio of the CFP hydrogel slightly decreased upon varying the PVA content and incorporating PC@MnO_2_ NPs; nevertheless, it remained above 950%, demonstrating its exceptional water‐absorption capacity. As shown in Figure 3B, all three hydrogels exhibited degradability, with degradation rates accelerating over time. Among them, the CFP/PC@MnO_2_ hydrogel displayed the slowest degradation rate, which is advantageous for maintaining structural and functional integrity at the wound site over an extended period. Investigating the drug release kinetics of hydrogels is critical for achieving sustained and controllable drug delivery. Given the elevated levels of ROS in the diabetic wound microenvironment, we simulated this pathological condition using PBS (pH 7.4) containing 0.5, 1.0, and, 2.0 mM H_2_O_2_ and evaluated the drug release behavior of the CFP/PC@MnO_2_ hydrogel. As illustrated in Figure 3C, the cumulative amount of released PC@MnO_2_ NPs from the CFP/PC@MnO_2_ hydrogel increased progressively over time. Moreover, under high‐ROS conditions, the release quantity was further enhanced, demonstrating a distinct ROS‐responsive release profile.

**FIGURE 3:**
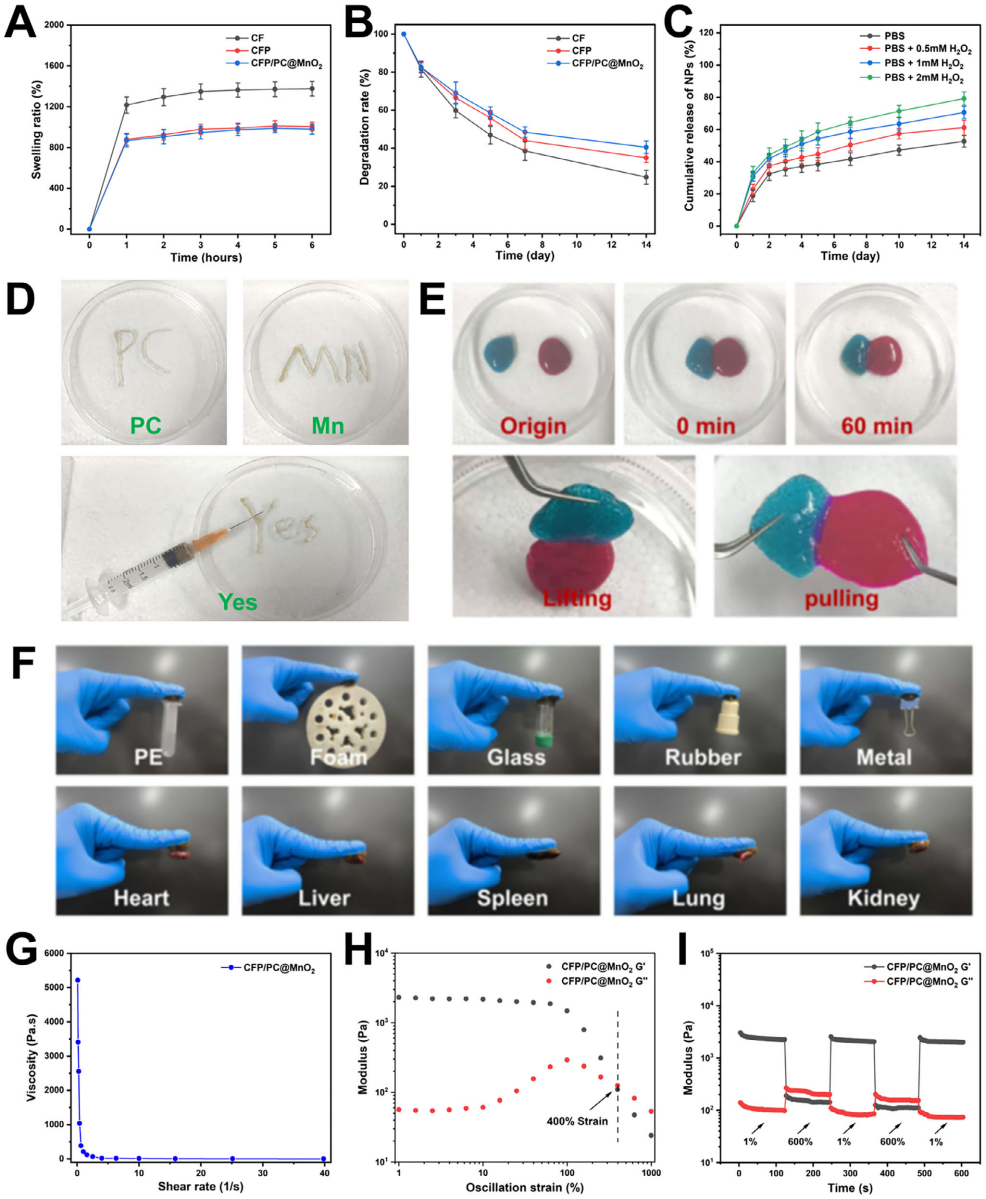
Physicochemical properties of CFP/PC@MnO_2_ hydrogel. (A) Swelling ratio of CF, CFP and CFP/PC@MnO_2_ hydrogel. Data are expressed as mean ± standard deviation. (*n* = 3). (B) Degradation experiments of CF, CFP and CFP/PC@MnO_2_ hydrogel. Data are expressed as mean ± standard deviation. (*n* = 3). (C) Release experiments of CFP/PC@MnO_2_ hydrogel under varying H_2_O_2_ concentrations. Data are expressed as mean ± standard deviation. (*n* = 3). (D) Injectable property of CFP/PC@MnO_2_ hydrogel. (E) Self‐healing property of CFP/PC@MnO_2_ hydrogel. (F) Adhesion effect of CFP/PC@MnO_2_ hydrogel. (G) Shear‐thinning property of CFP/PC@MnO_2_ hydrogel. (H) Rheological frequency sweep of CFP/PC@MnO_2_ hydrogel. (I) Continuous step strain test of CFP/PC@MnO_2_ hydrogel.

For irregular wound shapes, injectability, self‐healing, and adhesion of hydrogels are critical [50]. As shown in Figure 3D, the CFP/PC@MnO_2_ hydrogel could be extruded via syringe to form the letters “PC”, “Mn”, and “Yes”. As shown in Figure 3E, Rhodamine B and methylene blue staining demonstrated that the CFP/PC@MnO_2_ hydrogel exhibited excellent self‐healing ability: the interface became indistinct within 60 min and could withstand lifting tests, confirming its reversible dynamic bonding. In adhesion tests, this hydrogel exhibited robust adhesion to rat organs (heart, liver, spleen, lung, kidney) and diverse substrates (polyethylene, foam, glass, rubber, and metal) (Figure 3F). As shown in Figure 3G, the viscosity of the CFP/PC@MnO_2_ hydrogel decreased sharply with increasing shear rate, indicating excellent shear‐thinning injectability. Rheological characterization of the CFP/PC@MnO_2_ hydrogel was performed. As shown in Figure 3H, both the storage modulus (G′) and loss modulus (G″) remained stable up to 10% strain; beyond 400% strain, G′ and G″ intersect, signifying structural collapse and a solid‐to‐liquid transition. Cyclic step‐strain tests further evaluated the rheological recovery under high (600%) and low (1%) strains. Upon removal of high strain, both G′ and G″ recovered nearly to their initial values, confirming network rupture and subsequent self‐repair—demonstrating robust self‐healing capability (Figure 3I). In summary, the CFP/PC@MnO_2_ hydrogel exhibits suitable mechanical strength, swelling capacity, slow degradation, injectability, self‐healing, tissue adhesion, and ROS‐responsive drug release, which are key properties for diabetic wound treatment.

### 3.6 In Vitro Biocompatibility of CFP/PC@MnO_2_ Hydrogel

Good biocompatibility was a prerequisite for the application of hydrogels in wound repair. Hemocompatibility was evaluated using rat venous blood. As shown in Figure S10, compared with the positive control group, the CFP hydrogel, PC@MnO_2_ NPs, and the CFP/PC@MnO_2_ hydrogel exhibited hemolysis rates (HR) that were nearly identical to those of the negative control group, with all measured values not exceeding 5%. These results indicated that the CFP/PC@MnO_2_ hydrogel possessed excellent hemocompatibility. Subsequently, cellular compatibility was evaluated using L929 fibroblast cells and human umbilical vein endothelial cells (HUVECs). As shown in Figure S11, no significant cytotoxicity was observed after 24 h of co‐culture across the entire PC@MnO_2_ NPs concentration range (0–500 µg mL^−1^). Subsequently, the cytotoxicity of the CFP hydrogel and the CFP/PC@MnO_2_ hydrogel was comparatively evaluated. As presented in Figure 4A,B, following 48 h of co‐incubation, the cell viability of the CFP hydrogel, PC@MnO_2_ NPs, and the CFP/PC@MnO_2_ hydrogel was comparable to that of the control group, with all samples exhibiting cell viability exceeding 95%.

**FIGURE 4:**
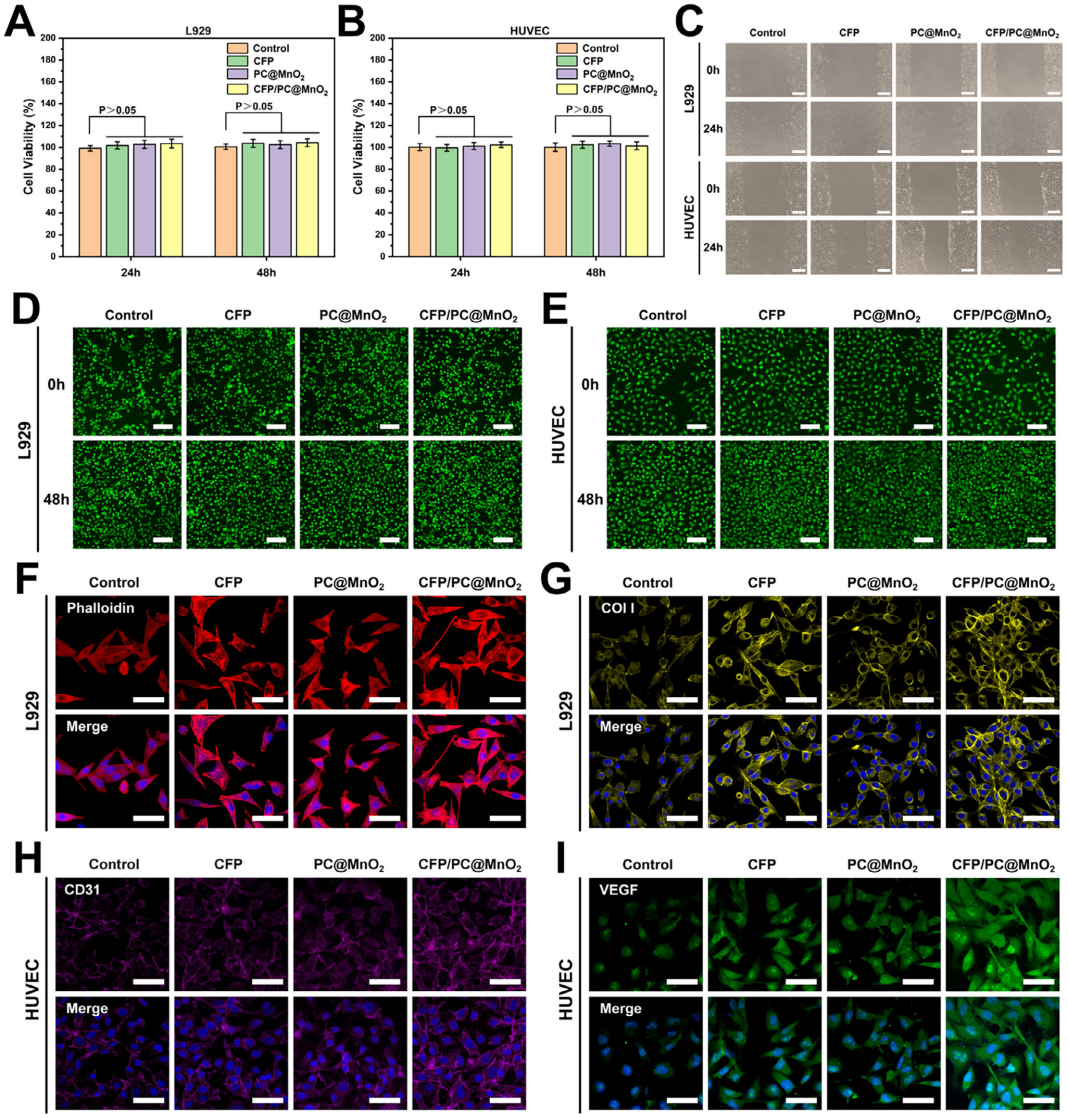
Biocompatibility and Bioactivity of CFP/PC@MnO_2_ hydrogel. (A,B) CCK‐8 results of L929 cells and HUVECs after different treatments. Data are expressed as mean ± standard deviation. (*n* = 6, one‐way ANOVA followed by Tukey's multiple comparison test, *p* > 0.05: not significant). (C) Images of L929 cell and HUVECs scratch and area treated with CFP/PC@MnO_2_ hydrogel after different treatments. Scale bar = 200 µm. (D,E) Cell Proliferation Assay of L929 cells and HUVEC. Scale bar = 200 µm. (F,G) Fluorescence images of cytoskeleton and Col I after different treatments. Scale bar = 50 µm. (H,I) Fluorescence images of cellular CD31 and VEGF expression. Scale bar = 50 µm.

### 3.7 In Vitro Bioactivity of CFP/PC@MnO_2_ Hydrogel

The effect of CFP/PC@MnO_2_ hydrogel on the migration and growth of L929 fibroblasts and HUVECs was investigated through scratch assays. After one day of incubation with different treatments, the extent of cell migration into the scratched areas varied across groups. Cells treated with CFP/PC@MnO_2_ hydrogel exhibited a significantly larger migrated area compared to the control group, demonstrating enhanced migratory capacity (Figure 4C; Figures S12 and S13). The effects of CFP/PC@MnO_2_ hydrogel extracts on the growth of L929 fibroblasts and HUVECs were further evaluated using live‐dead cell staining. As shown in Figure 4D,E, cells in all groups proliferated after 48 h of incubation. Compared with the control group, the CFP hydrogel group and the PC@MnO_2_ NPs group exhibited a positive effect on cell proliferation, with the most significant enhancement observed in the CFP/PC@MnO_2_ hydrogel group.

To further investigate the effects of hydrogels on the cytoskeleton of L929 cells, Phalloidin/DAPI cytoskeletal staining was performed after 3 days of culture. Compared with the control group, cells in all experimental groups exhibited well‐spread morphologies, and no significant reduction in the cytoplasmic area surrounding individual nuclei was observed, indicating that none of the treatments adversely affected cell adhesion or cytoskeletal spreading. Moreover, cells cultured on the CFP/PC@MnO_2_ hydrogel displayed a higher aspect ratio and larger spreading area than those cultured on PC@MnO_2_ NPs or the CFP hydrogel, suggesting that the CFP/PC@MnO_2_ hydrogel promotes cell adhesion and spreading on its surface, thereby facilitating tissue regeneration (Figure 4F). Considering that extracellular matrix (ECM) deposition constitutes a crucial phase in tissue regeneration, the influence of the hydrogels on collagen secretion by L929 fibroblasts was subsequently investigated. The findings demonstrated that cells across all treatment groups preserved a healthy morphology, and the secretion of type I collagen (Col I) was markedly increased in all treatment groups relative to the control group (Figure 4G). Notably, the CFP/PC@MnO_2_ hydrogel group exhibited the most pronounced pro‐collagen‐secretion effect. CD31 and VEGF served as critical pro‐angiogenic factors, playing pivotal roles in promoting endothelial cell migration and proliferation, as well as facilitating neovascularization. Immunofluorescence staining results demonstrated that both PC@MnO_2_ NPs and CFP hydrogel effectively enhanced VEGF expression. In comparison, the CFP/PC@MnO_2_ hydrogel further elevated the expression level of CD31 and VEGF (Figure 4H,I). The above results indicated that PC@MnO_2_ NPs and the CFP hydrogel collectively created a favorable microenvironment for cell proliferation, effectively maintained cytoskeletal architecture, and promoted ECM deposition and remodeling—thereby demonstrating enhanced potential in modulating the wound microenvironment.

### 3.8 Antioxidant and O2‐Producing of CFP/PC@MnO_2_ Hydrogel

Based on the excellent antioxidant and oxygen‐generating capabilities of PC@MnO_2_ NPs, the CFP/PC@MnO_2_ hydrogel also exhibited a significant capacity to scavenge free radicals, including ROS, as well as to release oxygen. To simulate an oxidative microenvironment, 0.1 mM H_2_O_2_ was used, and DCFH‐DA was employed as a fluorescent probe to detect intracellular ROS levels in L929 cells and HUVECs. The blank control group exhibited intense green fluorescence, whereas all other treatment groups showed reduced green fluorescence intensity. Notably, the CFP/PC@MnO_2_ hydrogel group exhibited the lowest fluorescence intensity, indicating its efficacy in reducing intracellular ROS (Figure 5A,B). Furthermore, intracellular hypoxia was assessed using the oxygen‐sensitive probe [Ru(dpp)_3_]Cl_2_. The blank control group displayed intense red fluorescence, whereas the treated groups showed diminished red fluorescence, with the CFP/PC@MnO_2_ hydrogel group exhibiting the weakest signal (Figure 5C,D). These observations suggest that the hydrogel effectively mitigates cellular hypoxia. Collectively, these results demonstrate that the CFP/PC@MnO_2_ hydrogel exhibits potent antioxidant and oxygen‐delivering capabilities, highlighting its potential as a therapeutic agent for wound healing and tissue regeneration. Moreover, studies have demonstrated that PC retains its intrinsic biological functional properties even after modification with metallic nanoparticles [28, 51]. To further investigate the bioactivity of PC in the PC@MnO_2_ NPs, an additional control group containing only free PC was established. Results showed that, at equivalent concentrations, free PC exhibited moderate antioxidant activity, whereas PC@MnO_2_ NPs displayed significantly enhanced antioxidant efficacy (Figure S14). This confirms that PC@MnO_2_ NPs not only preserve the inherent biological activity of phycocyanin but also synergistically augment its functional performance.

**FIGURE 5:**
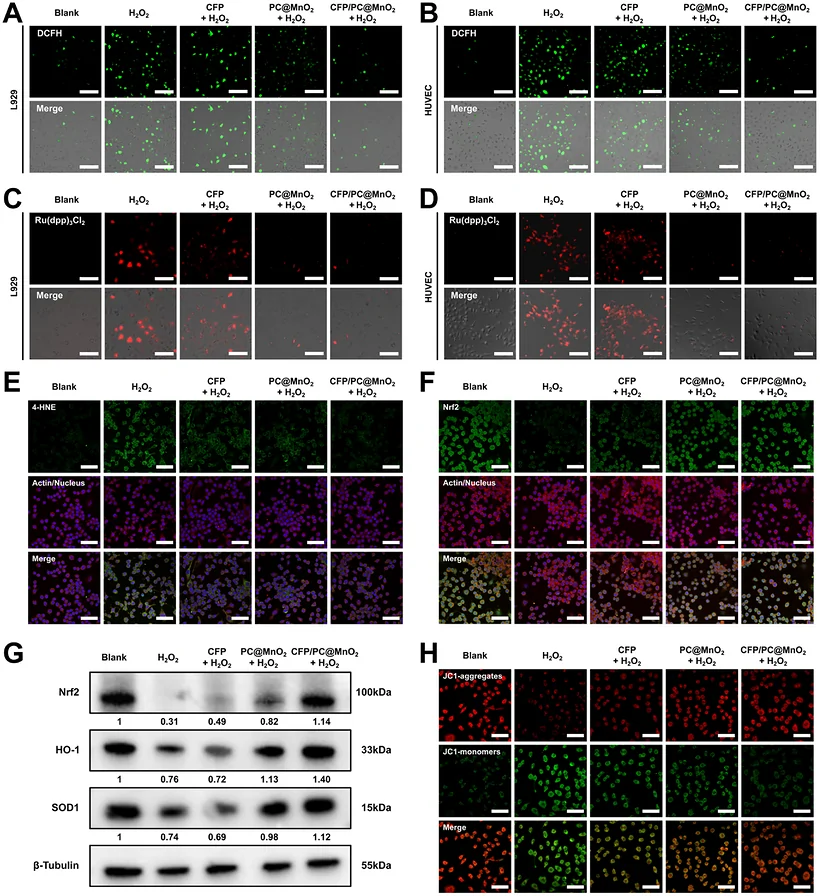
Antioxidant and Oxidative Stress–Reducing Capabilities of CFP/PC@MnO_2_ hydrogel. (A,B) Fluorescence images of L929 cells and HUVECs stained with DCFH‐DA after different treatments. Scale bar = 100 µm. (C,D) Intracellular O2 generation detection after different treatments. (E) Representative images showing 4‐HNE levels after different treatments. (F) Expression of Nrf2 protein after different treatments. (G) Western blot analysis of Nrf2, HO‐1, and SOD1 protein levels. (H) Mitochondrial membrane potential evaluation using the JC‐1 assay after different treatments. Scale bar = 50 µm.

Excessive ROS can inflict damage on biomacromolecules such as proteins, lipids, and nucleic acids, thereby mediating cellular injury while concurrently facilitating the production of highly reactive oxidative byproducts, including 4‐hydroxynonenal (4‐HNE) [52]. Consistent with this, intracellular levels of 4‐HNE were markedly elevated following hydrogen peroxide (H_2_O_2_) stimulation; however, this elevation was effectively attenuated by the combined application of the CFP hydrogel and PC@MnO2 NPs (Figure 5E). NF‐E2‐related factor 2 (Nrf2) serves as a principal transcriptional regulator of the cellular antioxidant defense mechanism. Under conditions of oxidative stress, Nrf2 is stabilized, evades proteasomal degradation, accumulates in the cytoplasm, and subsequently translocate to the nucleus [53]. Upon activation, Nrf2 exerts antioxidant effects by upregulating the expression of its downstream target protein heme oxygenase‐1 (HO‐1); this signaling pathway plays a pivotal role in oxidative stress–related diseases, suggesting that pharmacological modulation of this axis represents a promising therapeutic strategy [54]. To evaluate the activation effect of the CFP/PC@MnO_2_ hydrogel on antioxidant signaling pathways, researchers employed immunofluorescence staining and Western blot analysis. As shown in Figure 5F, exposure to hydrogen peroxide significantly downregulated Nrf2 expression. In contrast, all treatment groups exhibited a marked increase in green fluorescence intensity, with the CFP/PC@MnO_2_ hydrogel group showing the most pronounced effect. These results indicate that the hydrogel effectively activates the Nrf2‐mediated antioxidant stress response pathway, thereby counteracting hydrogen peroxide‐induced oxidative damage. Western blot analysis revealed that, compared with the control group, Nrf2 expression was significantly downregulated in the H_2_O_2_‐treated group. In contrast, both the PC@MnO_2_ NPs group and the CFP/PC@MnO_2_ hydrogel group effectively upregulated the expression of Nrf2, HO‐1, and SOD1. These findings indicate that H_2_O_2_ treatment suppresses the Nrf2‐mediated antioxidant signaling pathway, whereas the CFP/PC@MnO_2_ hydrogel robustly activates this pathway by enhancing the expression of these key antioxidant genes (Figure 5G and Figure S15).

Given the intricate interplay between oxidative stress and mitochondrial dysfunction in diabetic wound healing, excessive ROS could directly induce mitochondrial damage, thereby exacerbating oxidative stress [55]. Based on the pronounced efficacy of the CFP/PC@MnO_2_ hydrogel in suppressing oxidative stress and normalizing aberrant Nrf2 pathway activation, we further investigated its cytoprotective effects under conditions of elevated ROS. Mitochondria are critical organelles highly sensitive to ROS. To assess oxidative stress‐induced mitochondrial functional alterations, the fluorescent probe JC‐1 was employed [56]. As shown in Figure 5H, treatment with H_2_O_2_ significantly decreased mitochondrial red fluorescence while increasing green fluorescence in RAW 264.7 cells compared to the control group, indicating profound mitochondrial membrane depolarization. Conversely, all treatment groups exhibited a significant increase in red fluorescence alongside a marked reduction in green fluorescence, suggesting substantial attenuation of H_2_O_2_‐mediated mitochondrial damage. Notably, the CFP/PC@MnO_2_ hydrogel treatment group demonstrated the most pronounced protective effect. These results demonstrate that the CFP hydrogel and PC@MnO_2_ NPs act synergistically to mitigate H_2_O_2_‐induced mitochondrial dysfunction, thereby conferring robust cytoprotection.

In summary, the CFP/PC@MnO_2_ hydrogel demonstrates significant efficacy in mitigating oxidative stress and reestablishing cellular homeostasis by scavenging ROS, alleviating hypoxia, and protecting mitochondria, thereby showing promising potential as a clinically applicable wound‐healing material.

### 3.9 Immune and Inflammatory Regulation Mediated by the CFP/PC@MnO_2_ Hydrogel

An inflammatory model was established by stimulating RAW 264.7 macrophages with lipopolysaccharide (LPS), and the effects of different treatment groups on the expression of the pro‐inflammatory cytokine TNF‐α and the anti‐inflammatory cytokine IL‐10 were comparatively evaluated to assess the inflammatory regulatory capacity of the CFP/PC@MnO_2_ hydrogel. Both the CFP hydrogel and PC@MnO_2_ NPs effectively suppressed TNF‐α expression and enhanced IL‐10 expression. In comparison, the CFP/PC@MnO_2_ hydrogel group exhibited significantly more pronounced immunomodulatory effects (Figure 6A,B). These findings were further corroborated by RT‐qPCR analysis, which demonstrated that the CFP/PC@MnO_2_ hydrogel more potently inhibited mRNA expression of the pro‐inflammatory cytokine TNF‐α while concurrently upregulating mRNA expression of the anti‐inflammatory cytokine IL‐10 in macrophages (Figure 6C,D). To evaluate the effect of the CFP/PC@MnO_2_ hydrogel on macrophage polarization, we further assessed the expression levels of the M1 marker inducible nitric oxide synthase (iNOS) and the M2 marker CD206. The results showed that, compared with the blank control group, LPS stimulation significantly upregulated iNOS expression in RAW 264.7 macrophages, indicating polarization toward the pro‐inflammatory M1 phenotype. Conversely, interleukin‐4 (IL‐4) stimulation substantially elevated CD206 expression, reflecting polarization toward the anti‐inflammatory M2 phenotype. Treatment with either the CFP hydrogel or PC@MnO_2_ NPs resulted in a reduction of iNOS expression alongside an enhancement of CD206 expression. Notably, administration of the CFP/PC@MnO_2_ hydrogel elicited the most pronounced suppression of iNOS expression, accompanied by a significant increase in CD206 levels (Figure 6E,F). Subsequent quantitative flow cytometry analysis corroborated these findings, revealing that the CFP/PC@MnO_2_ hydrogel decreased the expression of the M1 polarization marker CD86 while simultaneously upregulating the M2 polarization marker CD206 (Figure 6G,H).

**FIGURE 6:**
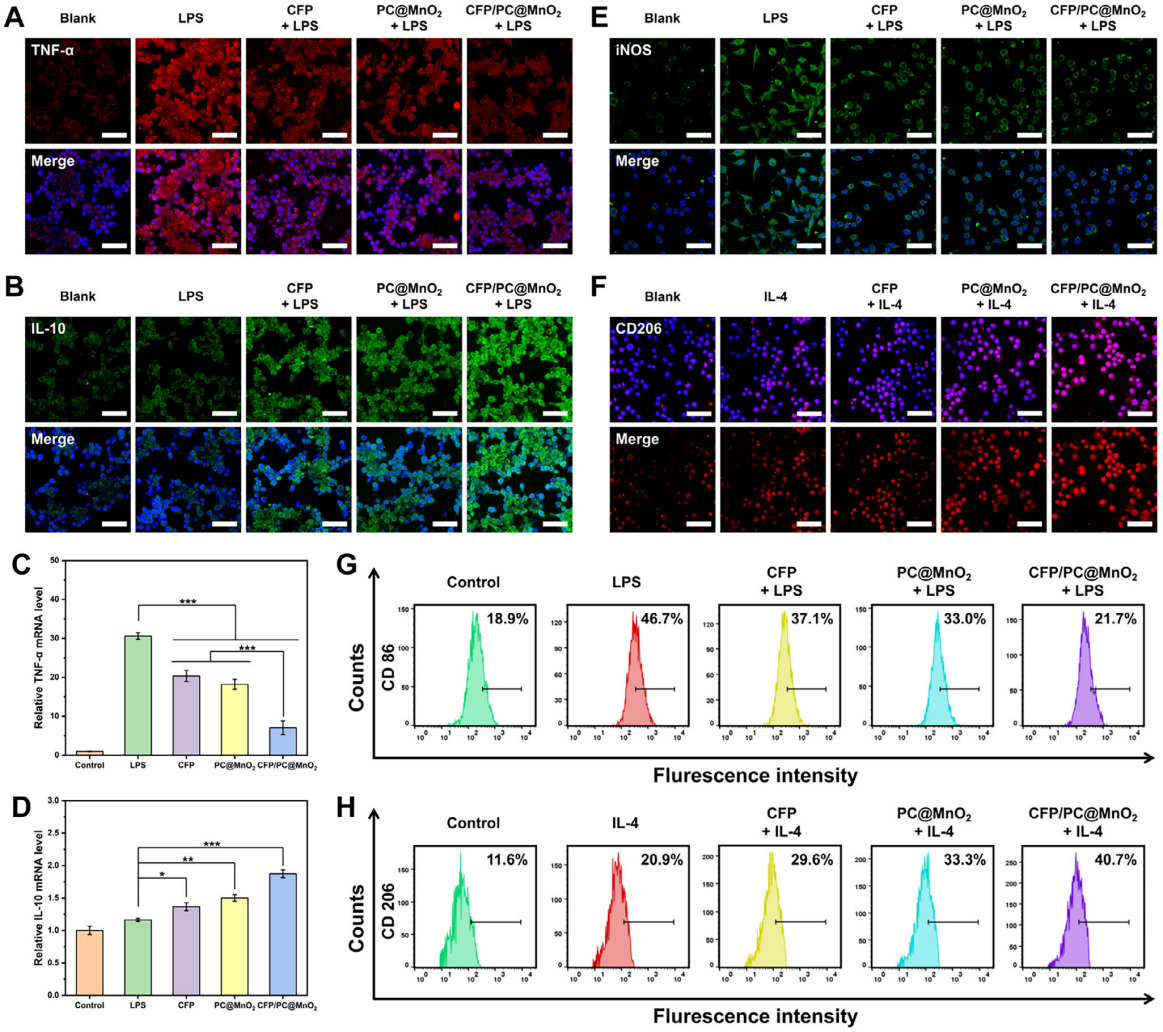
Immune and Inflammatory Regulation and Macrophage Phenotype Modulation by CFP/PC@MnO_2_ Hydrogel. (A,B) Immunofluorescence images of TNF‐α and IL‐10 across the different treatment groups. Scale bar = 100 µm. (C‐D) RT‐qPCR analysis of TNF‐α and IL‐10 gene expression in RAW 264.7 macrophages following different treatments under LPS stimulation. Data are expressed as mean ± standard deviation. (*n* = 3, one‐way ANOVA followed by Tukey's multiple comparison test, *p < 0.05, **p < 0.01, ***p < 0.001). (E,F) Immunofluorescence images of iNOS and CD206 across the different treatment groups. Scale bar = 100 µm. (G) Flow cytometry analysis of M1 macrophage marker CD86. (H) Flow cytometry analysis of M2 macrophage marker CD206.

These findings indicate that the CFP hydrogel synergizes effectively with PC@MnO_2_ NPs to modulate inflammatory responses and potently promote the phenotypic transition of macrophages from the M1 to the M2 state. Consequently, the CFP/PC@MnO_2_ hydrogel plays a pivotal role in facilitating M2 polarization and holds significant promise for accelerating diabetic wound healing.

### 3.10 In Vitro Antibacterial Effect of CFP/PC@MnO_2_ Hydrogel

Owing to the excellent photothermal conversion capability of PC@MnO_2_ NPs, the CFP/PC@MnO_2_ hydrogel retains a high photothermal conversion efficiency. PBS, the CFP hydrogel, PC@MnO_2_ NPs, and the CFP/PC@MnO_2_ hydrogel were separately irradiated with an 808 nm laser (2.0 W/cm^2^) for 10 min. As shown in Figure 7A,B, incorporation of PC@MnO_2_ NPs into the CFP hydrogel did not significantly impair its PTT efficacy. Given the prominent photothermal properties of CFP/PC@MnO_2_ hydrogel, evaluating its photothermal antibacterial efficacy against Gram‐positive *S.aureus* and Gram‐negative *E.coli* was essential. The antibacterial efficacy of PBS, CFP hydrogel, PC@MnO_2_ NPs, and CFP/PC@MnO_2_ hydrogel was comparatively evaluated under conditions with and without 808 nm laser irradiation (2.0 W/cm^2^). As shown in Figure 7C–F, in the absence of 808 nm laser irradiation, both the PC@MnO_2_ NPs group and the CFP/PC@MnO_2_ hydrogel group exhibited only modest antibacterial activity compared with the control group. Upon 808 nm laser irradiation, however, both the PC@MnO_2_ NPs group and the CFP/PC@MnO_2_ hydrogel group demonstrated a marked reduction in colony‐forming units (CFUs) of *S.aureus* and *E.coli*, achieving a photothermal antibacterial efficacy of up to 95%. Thus, the antibacterial mechanism of CFP/PC@MnO_2_ hydrogel is predominantly photothermal. Subsequently, bacterial samples subjected to different treatments were stained using the SYTO9/PI dual‐staining method. In the absence of 808 nm laser irradiation, only minimal red fluorescence was observed in both the PC@MnO_2_ NPs group and the CFP/PC@MnO_2_ hydrogel group. Upon 808 nm laser irradiation, however, both groups exhibited a marked increase in red fluorescence intensity compared with the control group, indicating a significant rise in the number of dead bacteria (Figure 7G,H). Given that diabetic wounds are highly susceptible to bacterial infection and subsequent biofilm formation, we further evaluated the biofilm‐eradication efficacy of the CFP/PC@MnO_2_ hydrogel. Crystal violet staining of bacterial biofilms revealed that, compared with the control group, treatment with either PC@MnO_2_ NPs or the CFP/PC@MnO_2_ hydrogel significantly reduced the biofilm coverage area of both *S.aureus* and *E.coli*. Notably, exposure to an 808 nm laser irradiation resulted in a notably greater reduction in biofilm area, demonstrating a substantially enhanced inhibitory effect of the hydrogel against biofilms formed by both bacterial strains (Figure 7I–L). Bacterial morphological changes across different treatment groups were further examined using SEM. As shown in Figure 7M,N, bacteria in the control group and those treated with the CFP hydrogel maintained intact morphology characterized by smooth surfaces and an absence of evident structural damage. In contrast, bacteria treated with PC@MnO_2_ NPs or the CFP/PC@MnO_2_ hydrogel displayed pronounced morphological alterations, including surface wrinkling, deformation, and cell wall disruption. These damaging effects were markedly intensified upon irradiation with 808 nm laser. Collectively, these findings underscore the potent photothermal antibacterial efficacy of both PC@MnO_2_ NPs and the CFP/PC@MnO_2_ hydrogel. During the photothermal conversion process of materials, localized oxidative stress may be exacerbated, thereby inducing adverse biological effects [57]. In contrast, MnO_2_ NPs possess intrinsic antioxidant nanozyme activity, which endows them with the capacity to mitigate oxidative stress and effectively alleviate cellular oxidative damage induced by photothermal therapy [58, 59, 60]. As shown in Figures S16 and S17, PC@MnO_2_ NPs exhibit intrinsic catalase‐ and superoxide dismutase‐mimicking activities, enabling in situ scavenging of ROS and concurrent generation of molecular oxygen—thereby effectively counteracting the adverse effects associated with photothermal therapy.

**FIGURE 7:**
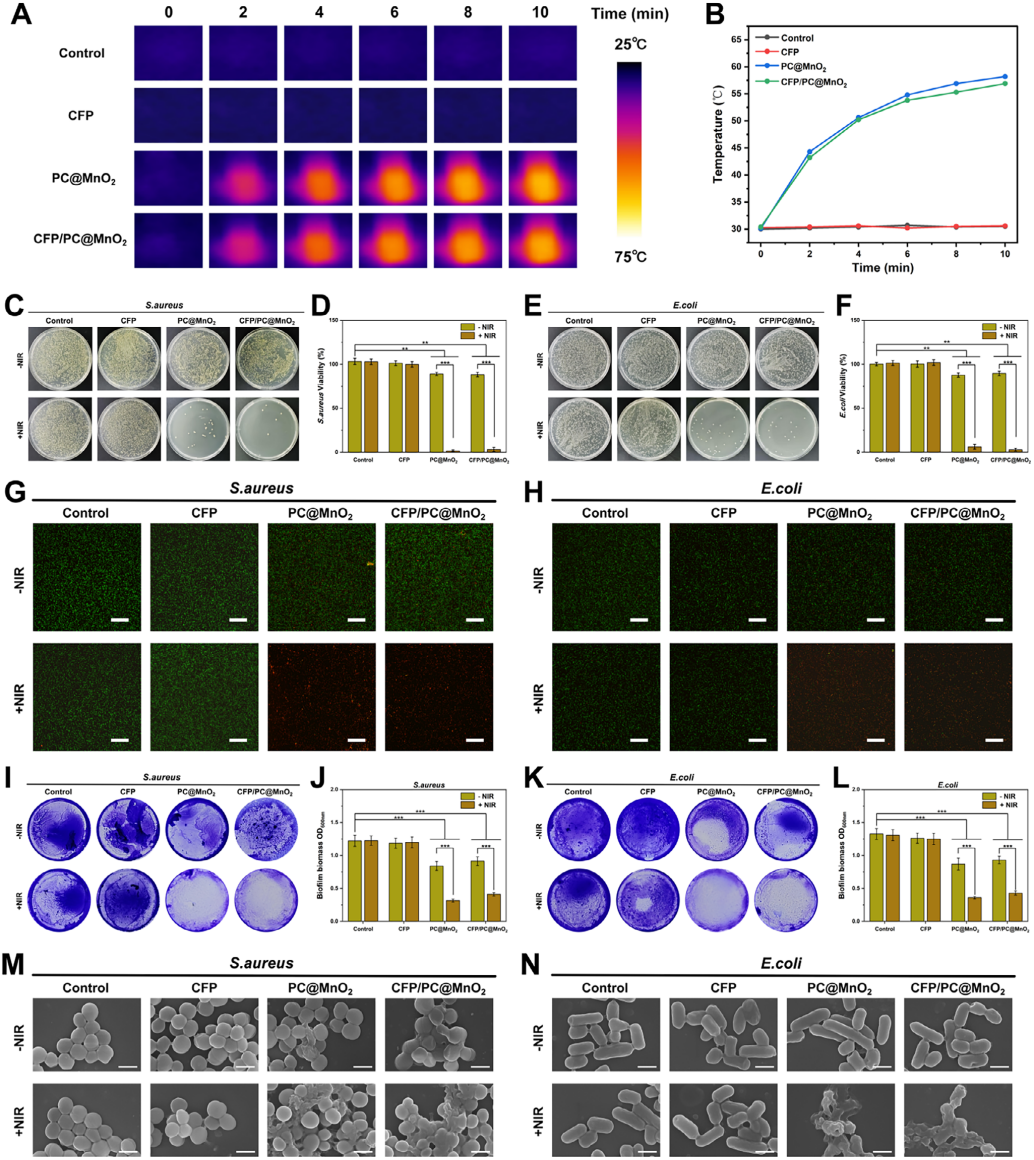
Antibacterial Properties of CFP/PC@MnO_2_ hydrogel. (A) In vitro photothermal images of Control, CFP hydrogel, PC@MnO_2_ NPs, CFP/PC@MnO_2_ hydrogel. (B) Temperature profiles of the Control, the CFP hydrogel, PC@MnO_2_ NPs, the CFP/PC@MnO_2_ hydrogel under 808 nm laser. (C) Photographs of *S. aureus* bacterial colonies after different treatments. (D) Statistical data of bacterial viability for *S. aureus*. Data are expressed as mean ± standard deviation. (*n* = 3, one‐way ANOVA followed by Tukey's multiple comparison test, **p < 0.01, ***p < 0.001). (E) Photographs of *E.coli* bacterial colonies after different treatments. (F) Statistical data of bacterial viability for *E. coli*. Data are expressed as mean ± standard deviation. (*n* = 3, one‐way ANOVA followed by Tukey's multiple comparison test, **p < 0.01, ***p < 0.001). (G,H) Live/Dead fluorescence co‐staining images of *S. aureus* and *E. coli* after various treatments, Scale bar = 100 µm. (I–L) Crystal violet staining of *S.aureus* and *E. coli* biofilms after various treatments. Data are expressed as mean ± standard deviation. (*n* = 3, one‐way ANOVA followed by Tukey's multiple comparison test, ***p < 0.001). (M‐N) SEM images of *S. aureus* and *E. coli* after various treatments, Scale bar = 1 µm. **p < 0.01; ***p < 0.001.

### 3.11 In Vitro Wound Healing in Skin Model With Diabetic Wound

The CFP/PC@MnO_2_ hydrogel exhibits a synergistic combination of antioxidant, anti‐inflammatory, immunomodulatory, and photothermal antibacterial properties, thereby effectively addressing the complex requirements inherent in diabetic wound healing. In this study, thirty male rats aged eight weeks were randomly allocated into five experimental groups: a PBS control group, a group treated with PC@MnO_2_ NPs, a group receiving the CFP hydrogel, a group treated with the CFP/PC@MnO_2_ hydrogel, and a group administered the CFP/PC@MnO_2_ hydrogel in conjunction with NIR irradiation. Diabetic rat models were induced via intraperitoneal injection of streptozotocin. Successful model establishment was confirmed when fasting blood glucose levels exceeded 16.7 mM, accompanied by characteristic polydipsia, polyphagia, and polyuria. Subsequently, six full‐thickness skin defects, each measuring 10 mm in diameter, were created on the rat dorsum, followed by administration of the corresponding treatment. The hydrogels were administered for a total duration of 15 days. In the photothermal therapy group, 808 nm laser irradiation (2.0 W/cm^2^) was delivered on days 0, 2, and 4. Wound healing rates were assessed on days 0, 3, 7, 10, and 15; bacterial burden at the wound site was quantified on day 4; and skin tissue samples were collected on days 7 and 15 for histological analysis (Figure 8A). Prior to therapeutic application, the photothermal efficacy of the CFP/PC@MnO_2_ hydrogel was assessed in situ using an 808 nm laser at 2.0 W/cm^2^, with temperature changes monitored via thermal imaging. Results indicated that after 10 min of irradiation, the wound temperature in the CFP/PC@MnO_2_ hydrogel‐treated group increased markedly from 37.4°C to 56.4°C (Figure 8B). Studies have demonstrated that maintaining a stable temperature of 50–55 °C during photothermal therapy effectively eradicates bacteria while minimizing irreversible damage to normal mammalian cells [61, 62]. Throughout the entire treatment course, no obvious tissue necrosis, erythema, edema, or exudate was observed at the in vivo wound site. These findings indicate that the employed photothermal therapeutic parameters achieve effective antibacterial activity while ensuring the safety of surrounding healthy tissues.

**FIGURE 8:**
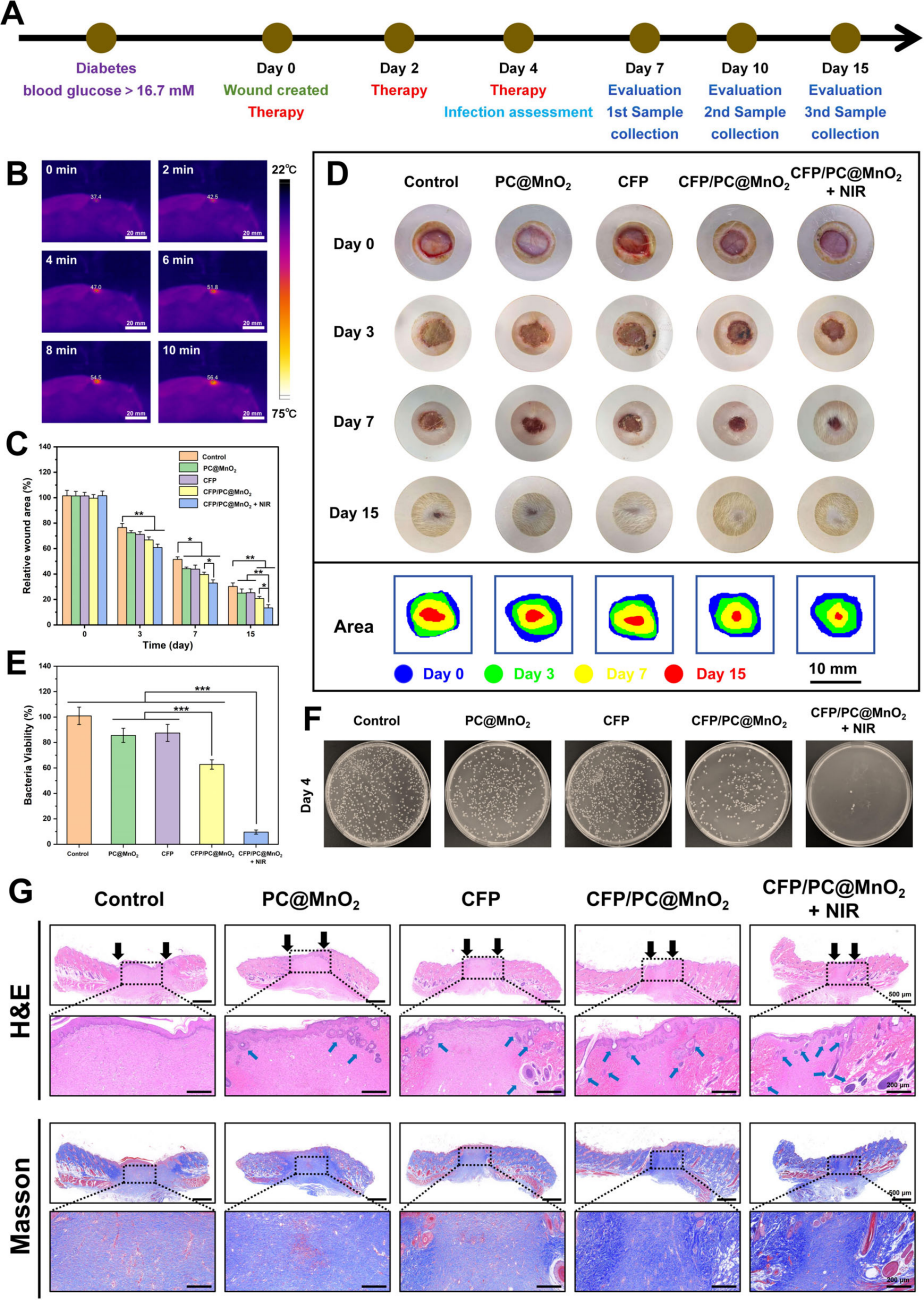
In Vivo Wound Healing Efficacy of the CFP/PC@MnO_2_ Hydrogel. (A) Schematic diagram of wound model establishment and treatment process. (B) Thermographic images showing temperature changes in wounds treated with the CFP/PC@MnO_2_ hydrogel. Scale bar = 20 mm. (C) Wound healing rates. Data are expressed as mean ± standard deviation. (*n* = 6, one‐way ANOVA followed by Tukey's multiple comparison test, *p < 0.05, **p < 0.01). (D) Representative photographs of diabetic wounds over 15 days. (E) Bacterial counts in the wound tissues on day 4 post‐treatment. (F) Representative photographs of bacterial colonies from wound tissues on day 4 post‐treatment. Data are expressed as mean ± standard deviation. (*n* = 3, one‐way ANOVA followed by Tukey's multiple comparison test, ***p < 0.001). (G) Representative H&E and Masson staining images in wound tissue on day 15, Scale bar bars: 500 µm (low magnification) and 200 µm (high magnification). Black arrow: dermal gap; dark blue arrow: hair follicle or sebaceous gland.

Throughout the entire treatment period, wound tissue size changes in each group were systematically assessed and recorded at predetermined time points. It was observed that the wound area in all groups gradually decreased over time. Compared with the control group, the therapeutic groups exhibited a significantly greater reduction in wound area, indicating a faster rate of wound healing (Figure 8C). Specifically, at the end of treatment, the wound area in the control group decreased to 30.2% of its initial size; in the PC@MnO_2_ NPs group, to 25.0%; in the CFP hydrogel group, to 25.4%; and in the CFP/PC@MnO_2_ hydrogel group, to 16.1%. Notably, the CFP/PC@MnO_2_ hydrogel group subjected to combined NIR irradiation exhibited the most pronounced reduction, with the wound area reduced to only 13.3% of its initial size (Figure 8D). To further validate the in vivo photothermal antibacterial efficacy of the CFP/PC@MnO_2_ hydrogel, bacterial colonization at the wound site was comparatively assessed on day 4. As shown in Figure 8E, bacterial residues were observed to varying degrees in both the control group and all treatment groups, with the most severe residual bacterial burden occurring in the control group. In contrast, almost no bacterial residues were detected in the CFP/PC@MnO_2_ hydrogel‐combined with NIR irradiation group. The above results show that the CFP/PC@MnO_2_ hydrogel effectively promote diabetic wound healing; combine with photothermal therapy, it synergistically enhance both infection prevention and wound closure.

### 3.12 Histological Evaluation of Regenerated Tissues

The therapeutic efficacy of different treatments on diabetic wound healing was evaluated through H&E and Masson's trichrome staining (Figure 8G). At 15 days post‐treatment, all experimental groups demonstrated the presence of an intact and thickened neo‐epidermis overlaying the granulation tissue. Notably, the groups treated with CFP/PC@MnO_2_ hydrogel and CFP/PC@MnO_2_ hydrogel combined with NIR irradiation exhibited pronounced neofollicular structures, marked fibroblast proliferation beneath the epidermis, and abundant, well‐organized collagen deposition. In comparison, the PC@MnO_2_ NPs and CFP hydrogel groups showed moderate histological improvements, whereas the control group displayed the least favorable healing outcomes, characterized by the absence of neofollicles at the wound center.

Given the superior performance of the CFP/PC@MnO_2_ hydrogel in facilitating wound structural remodeling, further investigation was conducted to elucidate its underlying mechanisms. Chronic diabetic wounds were characterized by a microenvironment marked by hyperglycemia and persistent, excessive inflammatory responses, frequently accompanied by pathological accumulation of ROS, which further exacerbated inflammation and impairs tissue regeneration. To evaluate the in vivo ROS‐scavenging efficacy and hypoxia‐ameliorating capacity of the CFP/PC@MnO_2_ hydrogel, dihydroethidium (DHE) staining was performed on wound tissues on day 7 post‐treatment to assess oxidative stress levels, and hypoxia‐inducible factor‐1α (HIF‐1α) immunostaining was conducted to quantify the degree of tissue hypoxia. As shown in Figure 9A and Figure S18, wound tissues from the control group exhibited intense DHE‐derived red fluorescence, indicative of substantial ROS accumulation in diabetic wounds. In contrast, all treatment groups demonstrated reduced ROS levels; notably, the CFP/PC@MnO_2_ hydrogel and CFP/PC@MnO_2_ hydrogel + NIR groups showed the lowest red fluorescence intensity, significantly diminished relative to the other groups. Consistent with the aforementioned trend, HIF‐1α immunofluorescence staining revealed intense red fluorescence in wound tissues of the control group, indicating severe tissue hypoxia. In contrast, HIF‐1α expression was reduced in all treatment groups; notably, the CFP/PC@MnO_2_ hydrogel group and the CFP/PC@MnO_2_ hydrogel + NIR group exhibited the weakest fluorescence intensity. These findings indicate that the CFP/PC@MnO_2_ hydrogel effectively scavenges excess ROS in the diabetic wound microenvironment, thereby alleviating oxidative stress and hypoxia‐induced tissue damage.

**FIGURE 9:**
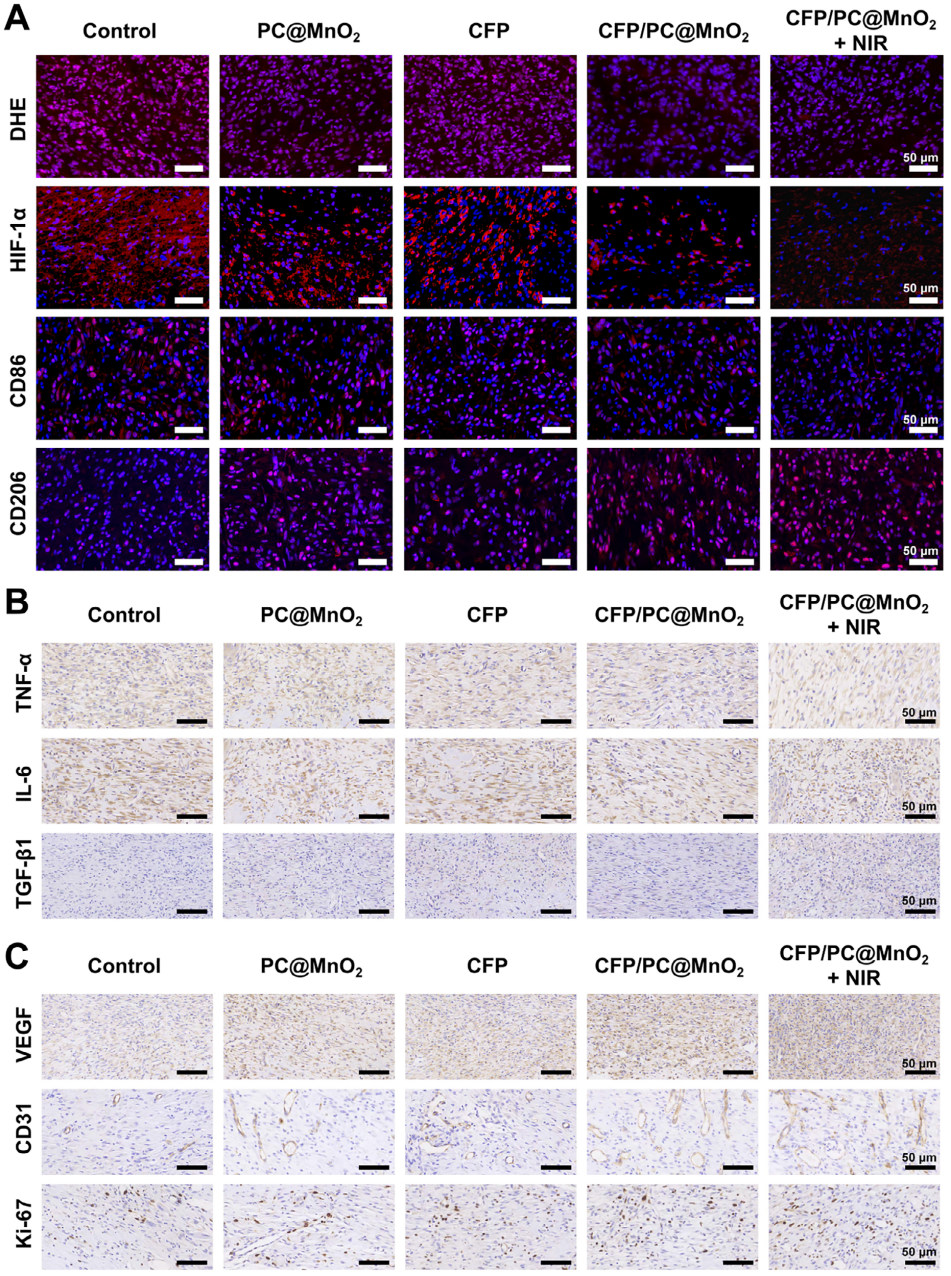
Oxidative Stress, Immune Modulation, Angiogenesis, and Tissue Proliferation in Diabetic Wound Healing. (A) Immunofluorescence staining of DHE, HIF‐1α, CD86, and CD206 in wound tissue on day 7. (B) Immunohistochemical staining images of TNF‐α, IL‐6, and TGF‐β1 in wound tissue on Day 7. (C) Immunohistochemical staining images of VEGF, CD31, and Ki‐67 in wound tissue on Day 10. Scale bar = 50 µm.

Immune regulation plays a pivotal role in wound healing, with macrophages functioning as principal mediators in the repair of diabetic wounds. In diabetic wounds, a sustained inflammatory microenvironment impedes the polarization of macrophages toward the M2 phenotype and disrupts the balance between M1 and M2 macrophage phenotypes, thereby obstructing effective healing. To investigate the regulatory effect of ROS scavenging on inflammation in diabetic wounds, macrophage polarization was evaluated through immunofluorescent staining targeting CD86 and CD206 markers. Results showed that the M1/M2 ratio was significantly reduced in both the CFP/PC@MnO_2_ hydrogel treatment group and the CFP/PC@MnO_2_ + NIR hydrogel treatment group, indicating that the ROS‐scavenging activity of these hydrogels effectively alleviates oxidative stress in diabetic wounds and promotes a shift in macrophage polarization toward the anti‐inflammatory M2 phenotype (Figure 9A and Figure S18). To further investigate the impact of macrophage phenotypic transition on the inflammatory microenvironment, the expression levels of M1‐associated pro‐inflammatory cytokines, tumor necrosis factor‐alpha (TNF‐α) and interleukin‐6 (IL‐6), as well as the M2‐associated tissue repair cytokine transforming growth factor‐beta 1 (TGF‐β1), were quantified in wound tissues. As shown in Figure 9B and Figure S19, at day 7 post‐treatment, the control group exhibited significantly elevated levels of TNF‐α and IL‐6 concomitant with markedly decreased TGF‐β1 expression, indicative of a prevailing pro‐inflammatory state. Conversely, all treatment groups demonstrated reduced expression of TNF‐α and IL‐6 alongside increased TGF‐β1 levels, with the CFP/PC@MnO_2_ hydrogel combined with NIR irradiation producing the most substantial modulation. Collectively, these results indicate that the CFP/PC@MnO_2_ hydrogel, particularly when synergistically applied with photothermal therapy, effectively suppresses pro‐inflammatory cytokine expression while enhancing anti‐inflammatory and pro‐reparative cytokine production, thereby exerting a significant anti‐inflammatory effect and promoting accelerated healing of diabetic wounds.

Given that resolution of inflammation was a prerequisite for transition of tissue into the proliferative phase, vascularization and cellular proliferation within wound tissues were further investigated. VEGF and CD31 are well‐recognized biomarkers for assessing angiogenesis, while Ki‐67 is a widely accepted marker indicative of cellular proliferative activity. As shown in Figure 9C and Figure S20, on day 10 post‐treatment, all therapeutic groups demonstrated increased VEGF expression, a higher number of CD31‐positive cells, and elevated Ki‐67‐positive cell counts in wound tissues relative to the control group. Importantly, the CFP/PC@MnO_2_ hydrogel combined with NIR irradiation exhibited the most significant pro‐angiogenic and pro‐proliferative effects, markedly surpassing both the CFP/PC@MnO_2_ hydrogel alone and other treatment modalities. Collectively, these results suggest that the CFP/PC@MnO_2_ hydrogel, especially when augmented by NIR stimulation, effectively enhances vascular endothelial cell formation and fibroblast proliferation, thereby promoting wound healing.

The results presented above demonstrate that the CFP/PC@MnO_2_ hydrogel, when utilized in conjunction with photothermal therapy, effectively mitigates oxidative stress and inflammatory responses in diabetic wounds. This is achieved through the inhibition of bacterial infection as well as the hydrogel's antioxidant and immunomodulatory properties. The combined multimodal mechanisms synergistically enhance angiogenesis and cellular proliferation. Therefore, this hydrogel system exhibits considerable promise as a therapeutic agent for the treatment of diabetic wound healing.

### 3.13 Transcriptomic Analysis of Wound Healing Acceleration Mechanisms

To deeply investigate the molecular mechanisms underlying accelerated diabetic wound healing mediated by the CFP/PC@MnO_2_ hydrogel in combination with NIR irradiation, RNA sequencing was performed on full‐thickness skin tissue samples harvested from wound sites of rats treated with CFP/PC@MnO_2_ hydrogel + NIR treatment group (*n* = 3) and control group (*n* = 3). Principal component analysis (PCA) revealed clear separation between the two groups in the principal component space, indicating substantial differences in their global gene expression profiles (Figure 10A). Comparative transcriptomic analysis identified a total of 2,878 differentially expressed genes (DEGs) between the CFP/PC@MnO_2_ + NIR treatment group and the control group, including 1,668 upregulated and 1,210 downregulated genes (Figure 10B,C). These findings demonstrate that this treatment strategy triggers broad molecular regulation at the transcriptional level. Gene Ontology (GO) enrichment analysis revealed that upregulated DEGs were predominantly associated with tissue regeneration, including processes such as cell–matrix adhesion, endothelial cell development, regulation of angiogenesis, and collagen fibril organization (Figure 10D). Conversely, the downregulated DEGs were predominantly enriched in pathways associated with oxidative stress and inflammatory response, further indicating that the combined treatment with CFP/PC@MnO_2_ hydrogel and NIR effectively ameliorated the inflammatory microenvironment in diabetic wounds (Figure 10E). In KEGG pathway enrichment analysis, upregulated DEGs were enriched in the cytokine–cytokine receptor interaction, ECM–receptor interaction, PI3K–Akt signaling pathway, and Wnt signaling pathways, collectively promoting endothelial cell proliferation, epithelial cell migration, and neovascularization to accelerate tissue repair. Meanwhile, downregulated DEGs were enriched in the NOD‐like receptor pathway and NF‐κB pathways, reducing the expression of immune‐inflammatory response–related genes and alleviating oxidative stress (Figure 10F). Gene Set Enrichment Analysis (GSEA) further revealed that gene sets associated with “tissue regeneration” (NES = 1.76) and “regulation of angiogenesis” (NES = 2.33) were significantly upregulated in the treatment group, whereas those linked to “regulation of inflammatory response” (NES = −2.13) and “cellular response to oxidative stress” (NES = −2.10) were significantly downregulated (Figure 10G). These results demonstrate that CFP/PC@MnO_2_ hydrogel + NIR treatment accelerated diabetic wound healing through a multi‐pathway cascade involving alleviation of oxidative stress, modulation of inflammation, enhancement of antioxidant defense, and promotion of tissue regeneration and angiogenesis.

**FIGURE 10:**
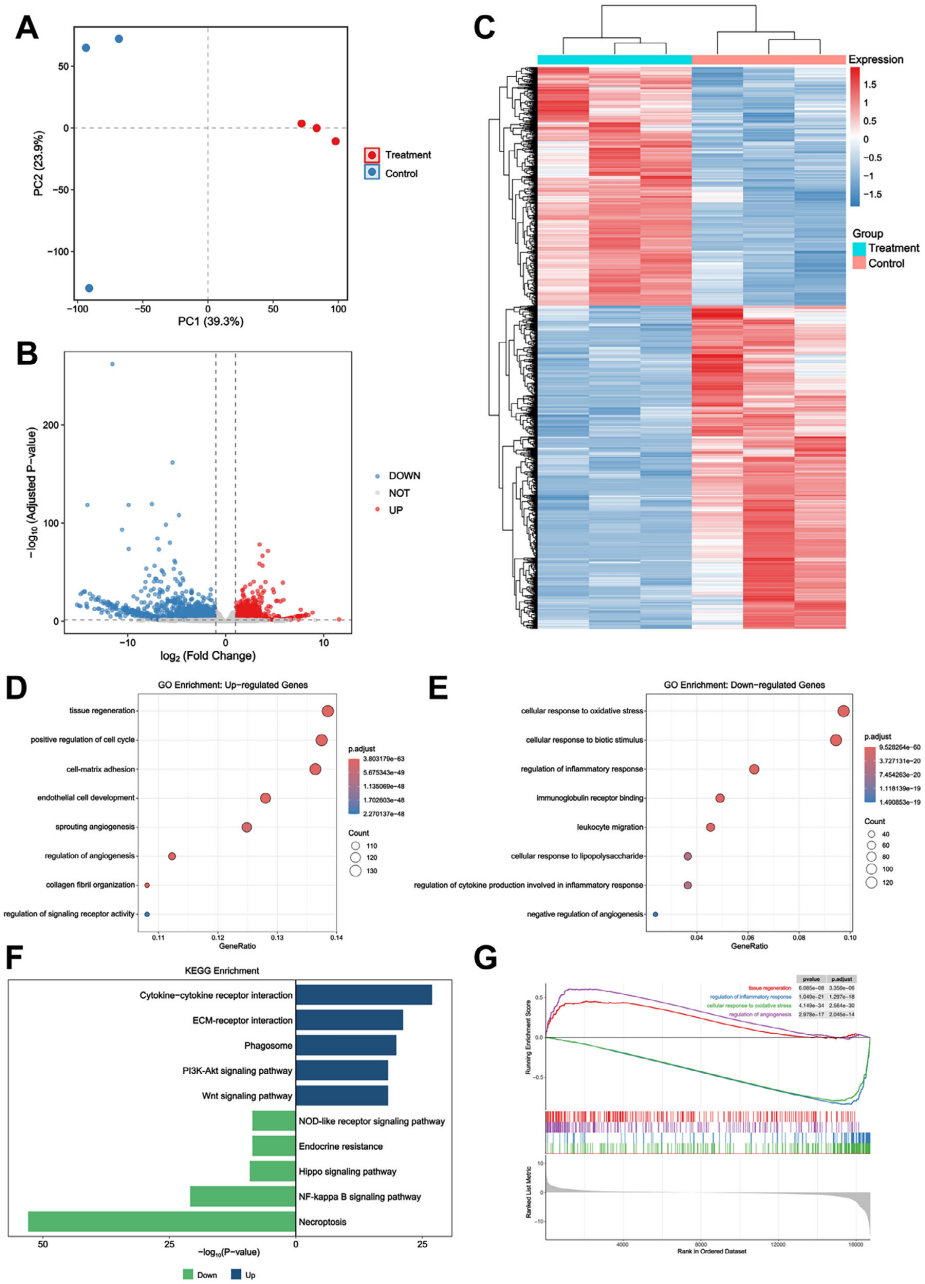
Transcriptomic analysis of potential mechanisms. (A) PCA plot of clustered datasets between the CFP/PC@MnO_2_ + NIR groups and Control groups. (B) Volcano plot of differentially expressed genes between groups. (C) Heatmap of differentially expressed gene clustering. (D,E) GO enrichment analysis for up‐DEGs and down‐DEGs. (F) KEGG pathway enrichment analysis for up‐DEGs and down‐DEGs. (G) GSEA of tissue regeneration, regulation of inflammatory response, cellular response to oxidative stress, and regulation of angiogenesis pathways.

### 3.14 Biosafety of CFP/PC@MnO_2_ Hydrogel

The CFP/PC@MnO_2_ hydrogel is applied topically to wounds and shows gradual degradation over 15 days as wound healing progresses (Figures S21 and S22). Throughout the 15‐day treatment period, the health status of all rats was meticulously monitored, with no notable changes in behavioral performance observed in any of the treatment groups. Following completion of the treatment, a comprehensive assessment of organ morphology and hematological parameters was performed for all subjects. Histopathological analysis using hematoxylin and eosin (H&E) staining revealed no significant tissue damage in the heart, liver, spleen, lungs, or kidneys of rats in any treatment group when compared to the control group (Figure S23). Furthermore, no clinically meaningful abnormalities were detected in routine hematological indices (WBC, RBC, PLT, Hb) and key serum biochemical markers (ALT, AST, CREA, ALB) (Figures S24 and S25). The CFP/PC@MnO_2_ hydrogel gradually degrades in the wound environment and exhibits good biocompatibility. Released Mn^2^^+^ is likely confined to the wound site, with minimal systemic exposure and efficient hepatic and renal clearance. Supported by literature on the biosafety of carboxymethyl chitosan‐based hydrogels and MnO_2_ NPs, we preliminarily assessed its long‐term toxicity risk as low [63, 64, 65]. Collectively, these findings demonstrate that the CFP/PC@MnO_2_ hydrogel exhibits favorable biocompatibility and biosafety.

## 4 Conclusions

In summary, this study constructs a functional hydrogel (CFP) via covalent cross‐linking of carboxymethyl chitosan (CMCS), 4‐formylphenylboronic acid (4FPBA), and polyvinyl alcohol (PVA) through Schiff base and boronate ester bonds. Subsequently, the CFP scaffold is integrated with phycocyanin‐modified manganese dioxide nanoparticles (PC@MnO_2_ NPs). CFP/PC@MnO_2_ hydrogel exhibits suitable mechanical strength, injectability, self‐healing, tissue adhesiveness, and ROS‐responsive drug release. In addition, this hydrogel synergistically integrates the advantages of the CFP hydrogel and PC@MnO_2_ NPs, exhibiting excellent biocompatibility, efficient ROS scavenging capability, alleviation of wound hypoxia and oxidative stress, and suppression of inflammation, thereby achieving precise targeting of the complex pathological features of the diabetic wound microenvironment. Furthermore, the integration of PC@MnO_2_ NPs imparts photothermal antibacterial functionality to the hydrogel, providing a robust approach for preventing wound infections. Both in vitro and in vivo studies reveal that, in conjunction with photothermal therapy, the CFP/PC@MnO_2_ hydrogel efficiently eliminates wound‐associated bacteria, modulates inflammatory processes, promotes M2 macrophage polarization, and significantly enhances angiogenesis and collagen deposition, ultimately accelerating wound healing in diabetic rat models. Collectively, the CFP/PC@MnO_2_ hydrogel represents a novel, multifunctional wound dressing that opens a promising therapeutic avenue for diabetic wound management.

## Author Contributions

**Zhi Xu**: Data Curation, Formal Analysis, Methodology, Writing – original draft Writing. **Xiaodong Luo**: Formal Analysis, Writing – Original Draft Writing. **Jingxian Wu**: Data Curation, Writing – Original Draft Writing. **Chaoyang Huang**: Methodology, Data Curation, Investigation. **Zhuoying Yang**: Data Curation, Investigation. **Yixiang HePeng**: Data Curation, Investigation. **Bosong Zhou**: Data Curation, Investigation. **Xiang Li**: Supervision, Validation, Writing – Review &Editing. **Ruiyuan Liu**: Conceptualization, Resources, Supervision, Validation, Writing – Review &Editing. **Xu Wu**: Conceptualization, Funding Acquisition, Resources, Supervision, Validation, Writing – Review &Editing.

## Ethics Statement

Animal experiments were conducted with approval from the Ethical Committee of Animal Experiments of Nanfang Hospital, Southern Medical University (IACUC‐LAC‐20260213‐001) and according to the Committee Guidelines.

## Conflicts of Interest

The authors declare no conflicts of interest.

## Supporting information




**Supporting File**: advs76774‐sup‐0001‐SuppMat.docx.

## Data Availability

The data that support the findings of this study are available on request from the corresponding author (Xu Wu) upon reasonable request. However, the raw sequencing data cannot be publicly deposited because they are jointly used by members of the GDNRC2024[27] project, and access requires prior approval from all project members.
