# Supplementary material for: Multifunctional Manganese Oxide Nanocomposite Hydrogel With Synergistic Reactive Oxygen Species‐Scavenging, Oxygen‐Generating, Immunomodulatory, and Photothermal Antimicrobial Activities for Enhanced Diabetic Wound Healing
Source: Adv Sci (Weinh). 2026 Jul 24;13(58):e76774. doi: 10.1002/advs.76774 (PMC13398130; doi:10.1002/advs.76774)
Supplement: Supplementary file 1 [file ADVS-13-e76774-s001.docx]

Supporting Information

**Multifunctional Manganese Oxide Nanocomposite Hydrogel with Synergistic Reactive Oxygen Species-Scavenging, Oxygen-Generating, Immunomodulatory, and Photothermal Antimicrobial Activities for Enhanced Diabetic Wound Healing**

Zhi Xu ^abc,1^, Xiaodong Luo ^c,1^, Jingxian Wu ^g,1^, [Chaoyang Huang](https://pubmed.ncbi.nlm.nih.gov/?term=Huang+C&cauthor_id=40822934) ^e^, Zhuoying Yang ^a^, Yixiang HePeng ^a^, Bosong Zhou ^a^, Xiang Li ^d,*^, Ruiyuan Liu ^f,*^, Xu Wu ^ab,*^

*^a^* Department of Thoracic Surgery, Nanfang Hospital, Southern Medical University, Guangzhou, 510515, China

*^b^* Huiqiao Medical Center, Nanfang Hospital, Southern Medical University, Guangzhou, 510515, China

^c^ Department of General Practice, Nanfang Hospital, Southern Medical University, Guangzhou, 510515, China

*^d^* Department of Emergency Medicine, Nanfang Hospital, Southern Medical University, Guangzhou, 510515, China

^e^ Department of Burns, Nanfang Hospital, Southern Medical University, Guangzhou, 510515, China

^f^ Biomaterials Research Center, School of Biomedical Engineering, Southern Medical University, Guangzhou, 510515, China

^g^ Cardiovascular Surgery, the Second Affiliated Hospital of Kunming Medical University, Kunming 650106, China

*Corresponding authors.

E-mail addresses: [wuxu_southhospital@163.com](mailto:wuxu_southhospital@163.com) (X. Wu), [ruiyliu@smu.edu.cn](mailto:ruiyliu@smu.edu.cn) (R. Liu), [li920402@smu.edu.cn](mailto:li920402@smu.edu.cn) (X. Li)

^1^These three authors contributed equally to this work.

**Materials**

Phycocyanin (C-PC), Carboxymethyl chitosan, 4-Formylphenylboronic acid and Manganese chloridewere were obtained from Macklin (China). 2,2'-azino-bis(3-ethylbenzothiazoline-6-sulfonic acid) diammonium salt (ABTS) and Polyvinyl Alcohol (PVA) were obtained from Aladdin (China). Superoxide Dismutase(SOD) Activity Assay Kit was obtained from ACMEC (China). The L929 cells, HUVECs cells and RAW264.7 cells were obtained from Southern medical university in China. Cell culture reagents, including Minimum Essential Medium (MEM), Dulbecco's Modified Eagle Medium (DMEM), penicillin G (100 U/mL), streptomycin (100 U/mL), and a 0.25% trypsin-0.53 mM EDTA solution, Fetal Bovine Serum were purchased from Gibco (Grand Island, USA). Horse serum was supplied by iCell Bioscience Inc. (China). Staphylococcus aureus and Escherichia coli were provided by Nanfang Hospital in China. The SYTO9-PI Live/Dead Bacteria Stain Kit was purchased from Tianjingsha (China). Streptozotocin (STZ) was obtained from Aladdin (China). 2’,7’-dichlorodihydrofluorescein diacetate (DCFH-DA) sourced from Beyotime (China). [Ru(dpp)3]Cl2 (luminescent oxygen sensor) was obtained from MKbio (China). The CCK-8 assay kit; 4’,6-diamidino-2-phenylindole dihydrochloride (DAPI); FITC-phalloidin; Calcein acetoxymethyl ester (Calcein-AM); and Propidium iodide (PI), all acquired from Dojindo Co., Ltd. in Japan. The primary antibodies, including iNOS, CD206, 4-HNE, Nrf2, JC-1, were acquired from Proteintech Group (China). Fluorescently labeled antibodies for flow cytometry, CD86 and CD206, were procured from BioLegend (USA). The antibodies for TNF-α, IL-6, TGF-β1, CD31, VEGF, Ki-67, DHE, HIF-1α, CD86, CD206 were purchased from Servicebio (China). Deionized water was generated in our laboratory and used in our study.

**Assessment of H_2_O_2_ scavenging activity**

This study assessed the CAT-like activities of PC@MnO_2_ NPs at concentrations of 0, 10, 20, 30, 40, and 50 μg/mL using a catalase assay kit (Beyotime, China), following the manufacturer’s instructions. After 30 min incubation, absorbance was measured for each sample.

**Assessment of O2•− scavenging activity**

O₂•⁻ scavenging capacity of PC@MnO_2_ NPs was assessed using a commercial SOD Activity Assay Kit (Solarbio, China). Following the manufacturer’s protocol, PC@MnO_2_ NPs were added to the working solution at concentrations of 0, 10, 20, 30, 40, and 50 μg/mL. Absorbance was measured at 450 nm using a microplate reader. O₂•⁻ scavenging rate (%) = [(A_SOD_ − A_Sample_)/A_SOD_] × 100%. A_SOD_ represents the OD value of untreated SOD, and A_Sample_ represents the OD value of SOD after adding PC@MnO_2_ NPs.

**Assessment of ABTS+· scavenging activity**

After mixing 7mM ABTS solution with 2.45 mM ammonium persulfate solution, incubate overnight at room temperature in the dark to activate ABTS+• free radicals. PC@MnO_2_ NPs (0, 10, 20, 30, 40, and 50 μg/mL) were added to the ABTS⁺• solution and reacted for 1 h. Absorbance was measured at 730 nm. ABTS+• scavenging efficiency (%) = [(A_ABTS+•_ − A_Sample_)/A_ABTS+•_ ] × 100%. A_ABTS+•_ represents the OD value of untreated ABTS+•, and A_Sample_ represents the OD value of ABTS+• after adding PC@MnO_2_ NPs.

**Mechanical strength test**

The swelling behavior of the hydrogels was assessed by immersing them in PBS (pH 7.4) at room temperature. Samples were removed and weighed at predetermined time points over 24 h. Swelling ratio (%) = (W_s_ - W_d_) / W_d_ × 100%., where W_s_ and W_d_ are the weights of the swollen and dried hydrogels, respectively. Hydrogel mechanical properties were evaluated via uniaxial compression on a universal testing machine. Cubic specimens (5 mm × 5 mm × 5 mm) were compressed at 1 mm/min, and Young’s modulus was calculated from the initial linear region (0–10% strain) of the stress–strain curve. The rheological properties of the hydrogel were characterized at 25℃ using a DHR-1 rheometer. Amplitude scan tests (1 Hz) were performed for different strains (0.1–1000 %). The self-healing property of the hydrogel was evaluated by measuring changes in G′ and G″ under alternating step strain cycles of 1 % and 600 % (1 Hz).Shear rate scans (0.1–40 1/s) were performed to study the shear thinning properties.

**In vitro degradation behavior of hydrogels**

Record the initial weight of the hydrogel as W₀. At predetermined time points, remove the samples and weigh them to obtain the weight at time (Wt). The remaining mass of the hydrogel was determined by the following equation: Remaining mass (%) =Wt/W₀ ×100%.

**In vitro drug release tests**

The release profile of PC@MnO_2_ NPs from the hydrogels was assessed by immersing 500 μL of CFP/PC@MnO_2_ hydrogel in 5 mL PBS (pH 7.4) or PBS (pH 7.4) containing 0.5mM, 1.0 mM and 2.0 mM H_2_O_2_. At predetermined time points, 1 mL of supernatant was withdrawn, filtered, and analyzed by UV-vis spectrophotometry to quantify released PC@MnO_2_ NPs. An equal volume of fresh PBS was then added to maintain constant total volume.

**Evaluation of Hemocompatibility**

RBCs isolated from rat venous blood were centrifuged at 1500 rpm for 15 min. Fifty microliters of a 5% RBC suspension was added to 500 μL of CFP hydrogel or 500 μL of CFP/PC@MnO_2_ hydrogel, respectively, in 1 mL saline. The mixtures were vortexed thoroughly and incubated for 2 h. Negative control: 0.9% NS only; positive control: distilled water. The supernatants were subsequently collected and analyzed using a microplate reader set to an absorbance wavelength of 540 nm. The hemolysis ratio of RBCs was calculated using the following equation: Hemolysis ratio percent =(A_sample_-A_negative_)/( A_positive_-A_negative_)×100%.

**Evaluation of Cytocompatibility**

L929 or HUVEC cells were seeded in 96-well plates at a density of 8 × 10³ cells per well. Cells were co-incubated with PC@MnO_2_ nanoparticles (0–500 μg/mL) for 24 h, and cell viability was assessed using the CCK-8 assay. Subsequently, cells were further incubated with either 50 μL of CFP hydrogel or 50 μL of CFP/PC@MnO_2_ hydrogel for 24 h or 48 h, and cell viability was again evaluated via the CCK-8 assay.

**Cell scratch assay**

The migratory capacity of L929 and HUVEC cells was evaluated via wound healing assay. Cells were seeded in 6-well plates (2 × 10^5^ cells/well) and cultured to 90–100% confluence. A linear wound was scratched across the center of each well using a sterile pipette tip; detached cells were removed by washing. Adherent cells were then incubated with medium containing CFP or CFP/PC@MnO_2_ hydrogel extracts. After 24 h, wound closure was imaged under an optical microscope and quantified using ImageJ. Mobility rate (MR) was calculated using the following formula:(S_0_-S_t_)/S_0_×100%. S_0_ is the initial scratch and S_t_ is the area of the healed scratch at each time point.

**Cell Proliferation Assay**

L929 or HUVEC cells were seeded into 96-well plates and cultured to a density of 4 × 10³ cells per well. Cells were then incubated with hydrogel extracts for 48 h, stained with Calcein-AM/PI in the dark for 30 min, and imaged under an inverted fluorescence microscope. The proliferative effect of the hydrogels was evaluated by Calcein-AM/PI staining.

**Cytoskeletal Staining**

Cytoskeletal morphology was evaluated via F-actin staining. L929 cells (2.0 × 10⁴ cells/well) were seeded into 24-well plates and subsequently exposed to PC@MnO_2_ NP or hydrogel extracts for 24 h. Following treatment, cells were fixed, then co-stained with phalloidin and DAPI, and finally visualized using fluorescence microscopy.

**Collagen Expression-Related Assays**

L929 cells were seeded in culture plates and treated with PC@MnO_2_ NPs or hydrogel extracts. After incubation, cells were fixed and stained with anti-Collagen I antibody and DAPI. Fluorescence images were acquired using a fluorescence microscope.

**Vascularization Assays**

HUVECs were seeded in culture plates and treated with either PC@MnO_2_ NPs or hydrogel extracts. After incubation, cells were fixed, stained with anti-CD31 and anti-VEGF antibodies, and counterstained with DAPI. Fluorescence images were acquired using a fluorescence microscope.

**Evaluation of 4-HNE Levels**

RAW 264.7 cells were seeded onto confocal dishes and cultured until reaching 80% confluence. Subsequently, 100 μL of hydrogel was added for pre-treatment for 24 h. Thereafter, oxidative stress was induced by supplementing the medium with 600 μM H₂O₂ and continuing incubation for 6 h. Following treatment, cells were fixed, permeabilized, and blocked. Immunofluorescent staining for 4-HNE was then performed to assess intracellular lipid peroxidation levels. Actin cytoskeletons were visualized using phalloidin staining, and nuclei were counterstained with DAPI. Images were acquired using a laser scanning confocal microscope.

**Assessment of Nrf-2 Nuclear Translocation**
 RAW 264.7 cells were seeded into confocal dishes and cultured until reaching 80% confluence. Subsequently, 100 μL of hydrogel was added for pre-treatment over 24 h. Thereafter, oxidative stress was induced by incubating the cells with 600 μM H₂O₂ for an additional 6 h. Following treatment, cells were fixed, permeabilized, and blocked. Immunofluorescent staining for Nrf-2 was then performed; actin cytoskeletons were visualized using phalloidin, and nuclei were counterstained with DAPI. Finally, nuclear translocation of Nrf-2 was assessed by laser scanning confocal microscopy.

**Mitochondrial Membrane Potential Assay**

JC-1 assay was used to evaluate the protective effect of hydrogels on mitochondrial membrane potential (ΔΨm) under H₂O₂-induced oxidative stress (600 μM). RAW 264.7 cells were seeded in 24-well plates and incubated until 80% confluency, then pre-treated with 100 μL hydrogel for 12 h. Cells were subsequently exposed to 600 μM H₂O₂ for 6 h. After incubation, cells were stained with JC-1 at 37°C for 20 min and imaged by fluorescence microscopy to assess ΔΨm changes.

**RT-qPCR**

Total RNA was extracted from RAW 264.7 macrophages using the EZ-press RNA Purification Kit and reverse transcribed into cDNA with the RevertAid First Strand cDNA Synthesis Kit. Quantitative real-time PCR (qPCR) was performed on a QuantStudio 6 Flex Real-Time PCR System using SYBR Premix Ex Taq. The thermal cycling conditions were: initial denaturation at 95°C for 10 min, followed by 40 cycles of 95°C for 15 s and 60°C for 1 min. A melt curve analysis was conducted to verify amplification specificity. Gene-specific primers were designed using NCBI Primer-BLAST, with GAPDH as the internal reference. Relative mRNA expression levels were calculated using the 2⁻ΔΔCt method. All experiments were independently repeated in triplicate.

**Western blot analysis**

Western blotting was used to assess Nrf2, HO-1, and SOD1 expression. Cells were lysed in RIPA buffer containing 1% protease and phosphatase inhibitors (per manufacturer’s instructions), and supernatants were collected. Protein concentration was determined by BCA assay. Equal amounts of protein were loaded, separated by SDS-PAGE, and transferred onto membranes via wet transfer. Membranes were blocked with 5% non-fat milk for 1 h. Primary antibodies were applied overnight at 4 °C. After three 10-min TBST washes, secondary antibodies were incubated under manufacturer-recommended conditions, followed by TBST washing. Immunoreactive bands were visualized using ECL reagent and quantified by ImageJ.

**RNA sequencing**

Wound tissue samples from rats in the control group and the CFP/PC@MnO_2_ hydrogel + NIR treatment group were collected for RNA sequencing analysis. Total RNA was extracted from wound tissues harvested on postoperative day 7 using TRIzol® reagent. RNA concentration and purity were quantified spectrophotometrically using a NanoDrop ND-1000 spectrophotometer (Thermo Fisher Scientific, Waltham, MA, USA), and RNA integrity was assessed electrophoretically using an Agilent 2100 Bioanalyzer (Agilent Technologies, Santa Clara, CA, USA). High-quality RNA samples were then processed for library construction and subjected to high-throughput sequencing on the Illumina NovaSeq™ 6000 platform (Illumina, Inc., San Diego, CA, USA). Differential expression analysis was performed using DESeq2 (|log₂FC| > 1, padj < 0.05). GO and KEGG enrichment analyses were conducted with clusterProfiler (p < 0.05).

**Figure S1.**The FT-IR spectra of PC and PC@MnO_2_ NPs.

**Figure S2.** The size of PC@MnO_2_ NPs after stored for 7 days in PBS. Data are expressed as mean ± standard deviation. (n = 3).

**Figure S3.** The size of PC@MnO_2_ NPs after stored for 7 days in DMEM. Data are expressed as mean ± standard deviation. (n = 3).

**Figure S4.** UV-vis detection of the scavenging ability of PC@MnO_2_ NPs at different concentration on ABTS⁺•.

**Figure S5.** The FT-IR spectra of CMCS, 4FPBA and PVA.

**Figure S6.** Gelation time of CFP hydrogel and CFP/PC@MnO_2_ hydrogel. Data are expressed as mean ± standard deviation. (n = 3, one-way ANOVA followed by Tukey’s multiple comparison test, ****p* < 0.001).

**Figure S7.** The SEM images of CFP hydrogel.

**Figure S8**. Compression stress-strain curves of CFP hydrogel.

**Figure S9.** Young’s modulus of CFP hydrogel. Data are expressed as mean ± standard deviation. (n = 3, one-way ANOVA followed by Tukey’s multiple comparison test, ****p* < 0.001).

**Figure S10.** Hemolytic performance after different treatments. Data are expressed as mean ± standard deviation. (n = 3, one-way ANOVA followed by Tukey’s multiple comparison test, ****p* < 0.001).

**Figure S11**. CCK-8 results of L929 and HUVEC treated with different concentrations of PC@MnO_2_ NPs. Data are expressed as mean ± standard deviation. (n = 6, one-way ANOVA followed by Tukey’s multiple comparison test, P＞0.05: not significant).

**Figure S12**.Statistics of L929 cells scratch area after different treatments. Data are expressed as mean ± standard deviation. (n = 3, one-way ANOVA followed by Tukey’s multiple comparison test, ***p* < 0.01, ****p* < 0.001).

**Figure S13**.Statistics of HUVECs scratch area after different treatments. Data are expressed as mean ± standard deviation. (n = 3, one-way ANOVA followed by Tukey’s multiple comparison test, ***p* < 0.01, ****p* < 0.001).

**Figure S14**. Fluorescence images of L929 cells and HUVECs stained with DCFH-DA after different treatments. Scale bar =100 μm.

**Figure S15**. Western blot analysis of Nrf2, HO-1, and SOD1 protein levels.

**Figure S16**. Intercellular ROS staining of L929 cells without and post-NIR irradiation. Scale bar =100 μm.

**Figure S17**. Intracellular O_2_ generation detection of L929 cells without and post-NIR irradiation. Scale bar =100 μm.

**Figure S18.** Quantitative analysis of DHE, HIF-1α, CD86 and CD206 relative area percentage. Data are expressed as mean ± standard deviation. (n = 3, one-way ANOVA followed by Tukey’s multiple comparison test, ***p* < 0.01, ****p* < 0.001).

**Figure S19.** Corresponding quantification of TNF-α, IL-6, and TGF-β1 expression. Data are expressed as mean ± standard deviation. (n = 3, one-way ANOVA followed by Tukey’s multiple comparison test, **p* < 0.05, ***p* < 0.01, ****p* < 0.001).

**Figure S20.** Corresponding quantification of VEGF, CD31, and Ki-67 expression. Data are expressed as mean ± standard deviation. (n = 3, one-way ANOVA followed by Tukey’s multiple comparison test, **p* < 0.05, ***p* < 0.01, ****p* < 0.001).

**Figure S21** Morphology of hydrogels in rats at different time points.

**Figure S22.** *In vivo* degradation rates of hydrogels at different time points. Data are expressed as mean ± standard deviation. (n = 3).

**Figure S23.** Major organs (heart, liver, spleen, lung, and kidney) after various treatments. Scale bar = 100 μm.

**Figure S24.** Blood routine examination of rats after various treatments. Data are expressed as mean ± standard deviation. (n = 6, one-way ANOVA followed by Tukey’s multiple comparison test, P＞0.05: not significant).

**Figure S25.** Blood panel analysis of rats after various treatments. Data are expressed as mean ± standard deviation. (n = 6, one-way ANOVA followed by Tukey’s multiple comparison test, P＞0.05: not significant).
